# NmFGF1-Regulated Glucolipid Metabolism and Angiogenesis Improves Functional Recovery in a Mouse Model of Diabetic Stroke and Acts *via* the AMPK Signaling Pathway

**DOI:** 10.3389/fphar.2021.680351

**Published:** 2021-05-07

**Authors:** Yeli Zhao, Shasha Ye, Jingjing Lin, Fei Liang, Jun Chen, Jian Hu, Kun Chen, Yani Fang, Xiongjian Chen, Ye Xiong, Li Lin, Xianxi Tan

**Affiliations:** ^1^ The First Affiliated Hospital of Wenzhou Medical University, Wenzhou, China; ^2^ School of Pharmaceutical Sciences, Wenzhou Medical University, Wenzhou, China; ^3^ Research Units of Clinical Translation of Cell Growth Factors and Diseases Research, Chinese Academy of Medical Science, Wenzhou, China

**Keywords:** ischemic stroke, diabetes, nmFGF1, angiogenesis, AMPK

## Abstract

Diabetes increases the risk of stroke, exacerbates neurological deficits, and increases mortality. Non-mitogenic fibroblast growth factor 1 (nmFGF1) is a powerful neuroprotective factor that is also regarded as a metabolic regulator. The present study aimed to investigate the effect of nmFGF1 on the improvement of functional recovery in a mouse model of type 2 diabetic (T2D) stroke. We established a mouse model of T2D stroke by photothrombosis in mice that were fed a high-fat diet and injected with streptozotocin (STZ). We found that nmFGF1 reduced the size of the infarct and attenuated neurobehavioral deficits in our mouse model of T2D stroke. Angiogenesis plays an important role in neuronal survival and functional recovery post-stroke. NmFGF1 promoted angiogenesis in the mouse model of T2D stroke. Furthermore, nmFGF1 reversed the reduction of tube formation and migration in human brain microvascular endothelial cells (HBMECs) cultured in high glucose conditions and treated with oxygen glucose deprivation/re-oxygenation (OGD). Amp-activated protein kinase (AMPK) plays a critical role in the regulation of angiogenesis. Interestingly, we found that nmFGF1 increased the protein expression of phosphorylated AMPK (*p*-AMPK) both *in vivo* and *in vitro*. We found that nmFGF1 promoted tube formation and migration and that this effect was further enhanced by an AMPK agonist (A-769662). In contrast, these processes were inhibited by the application of an AMPK inhibitor (compound C) or siRNA targeting AMPK. Furthermore, nmFGF1 ameliorated neuronal loss in diabetic stroke mice *via* AMPK-mediated angiogenesis. In addition, nmFGF1 ameliorated glucose and lipid metabolic disorders in our mouse model of T2D stroke without causing significant changes in body weight. These results revealed that nmFGF1-regulated glucolipid metabolism and angiogenesis play a key role in the improvement of functional recovery in a mouse model of T2D stroke and that these effects are mediated by the AMPK signaling pathway.

## Introduction

Ischemic stroke, a common cerebrovascular disease, is not only the main cause of death around the world, but is also the largest cause of long-term disability, thus representing a huge social burden. Diabetes increases the risk of stroke, increases related mortality, and delays recovery. According to statistics, the incidence of ischemic stroke in diabetic patients is significantly higher (by 2- to 6-fold) than non-diabetic stroke patients, and about 40% of patients with cerebral ischemia are associated with type 2 diabetes mellitus (Jouihan et al., 2013; Suda et al., 2015; Li et al., 2017; Jackson et al., 2020). Tissue plasminogen activator (tPA), the food and drug administration (FDA)-approved drug for stroke, is associated with a higher risk of hemorrhagic transformation (Jiang et al., 2020). Therefore, there is a critical need for explorative research aimed at discovering effective drugs to improve glucose and lipid metabolism as well as ameliorate cerebral ischemic injury in diabetic stroke.

One of the main characteristics of cerebral ischemia in diabetic patients is that the brain damage is aggravated by blood vessel injury. Angiogenesis in the ischemic border zone (IBZ) is a common endogenous repair response to cerebral ischemia and hypoxia, and has been shown to be positively correlated with the recovery of neurological function (Zhang et al., 2010; Banerjee et al., 2014; Rust et al., 2019). Therefore, angiogenesis is crucial to the treatment of brain injury after diabetic stroke. Endothelial cells play a primary role in angiogenesis but are functionally impaired in patients suffering from diabetic stroke (Yasmeen et al., 2019). Thus, the improvement of endothelial function will be an efficient method with which to encourage angiogenesis.

Fibroblast growth factor 1 (FGF1), a member of the FGF family, is abundantly distributed in the brain. FGF1 plays a significant role in neurogenesis and angiogenesis. It was reported that FGF1 promotes neurogenesis and angiogenesis in a mouse model of ischemic stroke and oxygen-glucose deprivation (OGD)-induced human brain microvascular endothelial cells (HBMECs) (Cheng et al., 2011; Wu et al., 2016; Zou et al., 2020). Furthermore, FGF1 can act as an insulin sensitizer to effectively reduce hyperglycemia in diabetes without adverse reactions (Scarlett et al., 2016; Gasser et al., 2017; Tennant et al., 2019). However, there have only been a few studies on FGF1-regulated angiogenesis and hyperglycemia in diabetic stroke. In addition, wild-type FGF1 increases the risk of tumorigenesis because of its mitogenic activity. However, non-mitogenic FGF1 (nmFGF1), derived from the loss of residues 1–27 in the N terminal of wild-type FGF1, does not promote cell proliferation. Therefore, the present study aimed to investigate the effects of nmFGF1 on angiogenesis and hyperglycemia in a mouse model of diabetic stroke.

Amp-activated protein kinase (AMPK), a major energy receptor and metabolic regulator, is one of the therapeutic targets for metabolic diseases and vascular diseases (Lu et al., 2019). Metformin, a commonly used AMPK activator, has been shown to improve post-stroke angiogenesis and recovery after experimental stroke (Jin et al., 2014; Venna et al., 2014). It has also been reported that adiponectin improves the function of endothelial cells by activating the AMPK pathway (Chen et al., 2009). Moreover, the activation of AMPK was shown to mediate the protection of fibroblast growth factor 1 from non-alcoholic fatty liver disease in mice by improving lipid metabolism (Lin et al., 2020). These findings suggested that AMPK may be involved in the protective effect of nmFGF1 on stroke in type 2 diabetic (T2D) mice and acts by regulating glucolipid metabolism and angiogenesis. However, only a few studies have investigated FGF1-regulated glucolipid metabolism and angiogenesis *via* the AMPK pathway in a mouse model of diabetic stroke. Therefore, we investigated whether nmFGF1-regulated glucolipid metabolism and angiogenesis *via* the AMPK pathway participated in the improvement of functional recovery in a mouse model of T2D stroke. We found that nmFGF1 treatment improved glucolipid metabolism in a mouse model of T2D stroke and enhanced angiogenesis *via* the AMPK signaling pathway. We also provide evidence that nmFGF1-modulated changes in a mouse model of T2D stroke contribute to functional recovery, at least in part.

## Materials and Methods

### Reagents and Antibodies

NmFGF1 was extracted and purified from *Escherichia coli* as described previously (Wu et al., 2005). The AMPK inhibitor (compound C), AMPK agonist (A-769662), along with primary antibodies against eNOS (No. ab66127), CD31 (No. ab28364), and β-actin (No. ab8227) were purchased from Abcam (Cambridge, MA, United States). Primary antibodies against *p*-eNOS (No. 9571s) and *p*-AMPKα (Thr172) (D4D6D) (No. 2535s) were purchased from Cell Signaling Technology (Danvers, MA, United States). Primary antibodies against MMP9 (No. IM09L), AMPK (No. 10929-2-AP), and MMP2 (No. WL03224) were purchased from Calbiochem (Merck, Darmstadt, Germany), Proteintech Group (Hubei, China), and Wanleibio (Liaoning, China), respectively. Primary antibodies against VEGF (No. sc-7269) and MAP2 (No. sc-74421), along with horseradish peroxidase-conjugated secondary antibodies were obtained from Santa Cruz Biotechnology (Dallas, TX, United States). Methylthiazolyldiphenyl-tetrazolium bromide (MTT) was purchased from Sigma (Sigma-Aldrich, St. Louis, MO, United States) for the colorimetric cell growth assay.

### Induction of Experimental Diabetes

C57BL/6 mice were purchased from the Animal Center of the Chinese Academy of Sciences (Beijing, China). All animal experiments were performed following approval of the Animal Research Ethics Committee of Wenzhou Medical University and were in accordance with the National Institutes of Health guidelines concerning the care and use of laboratory animals. Mice were raised in a constant temperature with 12 h light/dark cycles and allowed free access to food and water.

Type 2 diabetes mellitus (T2D) was induced *via* the combination of a high-fat diet (HFD) and a low dose of streptozotocin (STZ) (Liu et al., 2019). Mice aged 6–7 weeks were fed a rodent HFD (in which 60% of calories were derived from fat, Research Diets, New Brunswick, NJ, United States) for 12 weeks, and body weight was measured once a week. Diet-induced obese mice were injected (intraperitoneally, i. p.) with a low dose of STZ (35 mg/kg in 0.1 M citrate buffer, pH 4.5) (MilliporeSigma, Burlington, MA, United States) for five consecutive days. Mice with a fasting blood glucose level above 16.7 mmol/L were considered to be diabetic and used for further studies.

### Measurements of Changes in Body Weight and Biochemical Indicators

Type 2 diabetic mice are known to exhibit specific characteristics, including weight loss, hyperglycemia, and hyperlipidemia. To verify the successful induction of the diabetic model, we measured the levels of body weight, fasting blood glucose, triglyceride, and cholesterol, in our experimental mice. Two weeks after STZ injection, the blood glucose level was determined with a blood glucose meter (Abbott Diabetes Care Ltd., Doncaster, VIC, Australia). The serum levels of triglyceride (TG) and total cholesterol (T-CHO) were measured by commercial detection kits according to the manufacturer’s instructions (Nanjing Jiancheng Bioengineering Institute, Jiangsu, China).

In addition, the levels of glycated hemoglobin and insulin are considered to be very important detection indicators for diabetes. The concentrations of glycated hemoglobin A1c (HbA1c) and insulin were determined 7 days after the administration of nmFGF1, with a1cNow + meter (PTS Diagnostics, Whitestown, IN, United States) and an enzyme-linked immunosorbent assay (ELISA) detection kit (Nanjing Jiancheng Bioengineering Institute, Jiangsu, China), respectively.

### Photothrombotic Stroke and Drug Administration

Diabetic mice were fixed in a head stereotaxic frame, anesthetized with 4% choral hydrate (i.p.) (10 ml/kg), and a mid-slit incision was used to fully expose the skull. Mice were injected (i.p.) with rose bengal (30 mg/kg, Sigma-Aldrich, St. Louis, MO, United States) 5 min prior to illumination. A cold light source (KL1600 LCD, SCHOTT, Zeiss, Germany) was then placed on the surface of the skull (0 –3 mm lateral and 2.5 mm to −2.5 mm anterior to the bregma). A hollow black rubber pad was placed between the fiber head and the skull to prevent light exposure. After 15 min of brain illumination, the surgical incision was sutured immediately and the mice were placed into a postoperative incubator until they recovered from the anesthesia. Next, 24 h after stroke, the mice were then administered (i.p.) with nmFGF1 (0.25 mg/kg, 0.5 mg/kg, 0.75 mg/kg) or saline; these doses were then given every other day for up to 28 days.

### Infarction Assessment

Infarct size was measured by hematoxylin and eosin staining (HE, Beyotime Institute of Biotechnology, Shanghai, China) 7 days after the administration of nmFGF1. Specimens of brain tissue were fixed in 4% paraformaldehyde, embedded in paraffin, and then stained with HE. The infarct size was then imaged with a light microscope (Nikon, Tokyo, Japan) and analyzed with ImageJ software (NIH, Bethesda, Maryland, United States).

### Neurobehavioral Evaluation

Deficits in the neurological function of experimental mice were assessed on days 1, 3, 7, 14, 21, and 28, after stroke. These assessments involved the modified neurological severity scale (mNSS), the adhesive removal test, the grip strength test, and the foot fault test. The mNSS evaluates the somatosensory function of mice from the aspects of movement, sensation, balance and reflex (Li Z. et al., 2020). The mNSS score ranges from 0–18 points; higher values indicate a more severe functional impairment. The adhesive removal test is a well-established method for measuring sensorimotor deficit (Begum et al., 2018). Mice were kept in a clean cage for 1 min to acclimate to the environment. Then, round tapes (0.6 cm) were placed on the front paw of the mice. The time taken for the mice to remove these tapes was then recorded with a maximum of 300 s after the mice had been returned to the cage. The foot fault test was conducted as described previously (Guo et al., 2018). Mice were placed on a grid (60 cm × 40 cm, lattice diameter: 2 × 2 cm, 50 cm above the ground); a fault was defined as when the left forelimb fell into the grid. We also calculated the proportion of faults as a percentage of the total number of steps taken. Grip strength was measured with a grip strength meter (Ugo Basile, Comerio, Varese, Italy) three times; we then calculated the mean grip strength. The Morris water maze is an effective method to detect the spatial learning and memory ability of mice (Diederich et al., 2014). In brief, mice were trained for five consecutive days before recording escape latency with a video camera attached to the ceiling of the cage. On the sixth day of the test, mice performed a probe test with the platform removed. Platform-site crossings, swimming distance, and speed traveled in the target quadrant, were recorded and analyzed with a DigBhv animal behavioral analysis system.

### Vascular Density Analysis

Cerebral vessel density was measured by ink-gelatin perfusion (Hasan et al., 2013; Xue et al., 2014; Sutter et al., 2017). Mice were anesthetized with 4% chloral hydrate 7 days after the administration of nmFGF1. The mice then received an injection of 20 mL of saline into the left ventricle before being perfused with 10 mL of 2% ink-gelatin mixture (preheated to 37°C). After perfusion, mice were placed with their ventral side facing upwards at 4°C for 1 h to solidify the ink-gelatin. The mice were then sacrificed, and the brain tissue was removed. Finally, we investigated the morphology of the brain tissue, and the cerebral vessel density, with a camera (Cannon, Tokyo, Japan).

### Laser Doppler Flowmetry

Mice were anesthetized with 4% chloral hydrate and fixed in a stereotactic frame. Blood perfusion was then measured in each mouse over two random fields of the ischemic regions using a laser Doppler fiber (Oxford Optronix, Oxford, United Kingdom) at 0, 7, and 14 days post-injury.

### Western Blotting

Mice were sacrificed after anesthesia and the peri-infarct cortical tissue was harvested for analysis. Samples were lyzed on ice with protein extraction reagents (Beyotime Institute of Biotechnology, Shanghai, China) for 1 h, and then centrifuged at 12,000 rpm at 4°C for 10 min. The supernatant was then removed and stored at −20°C. Protein concentrations were quantified by the Bradford protein assay kit (Bio-Rad, California, United States). Samples were added in 5 × loading buffer and boiled for 10 min for denaturation. Forty micrograms of the total protein in each sample was then separated on 10% sodium dodecyl sulfate polyacrylamide gel electrophoresis (SDS-PAGE) gels, and then transferred to polyvinylidene fluoride (PVDF) membranes. The membranes were blocked with 5% non-fat milk in tris buffered saline with Tween 20 (TBST) at room temperature for 90 min and then incubated with the primary antibodies against VEGF, *p*-eNOS, eNOS, MMP9, MMP2, *p*-AMPK, AMPK, and β-actin, at 4°C overnight. The membranes were washed three times with TBST, and then incubated with horseradish peroxidase-conjugated secondary antibodies at room temperature for 1 h. The protein blots were detected by enhanced chemiluminescence (ECL) reagents (Biological Industries, Beit Hemek, Israel).

### EdU Administration

To evaluate angiogenesis, mice were injected (i.p.) with 5-ethynyl-2′-deoxyuridine (EdU, ST067, Beyotime, Shanghai, China) (50 mg/kg) on days 5, 6, and 7 following injury. EdU incorporation was then assessed using the BeyoClickTM EdU-488 kit (C0071S, Beyotime, Shanghai, China) on 5 µm coronal sections with immunofluorescence.

### Immunofluorescence Staining

Immunofluorescence staining was conducted to detect blood vessel density; the methodology for this has been described previously (Zou et al., 2020). In brief, mice were first anesthetized with chloral hydrate and then perfused with saline and 4% paraformaldehyde (PFA, 0.1 M phosphate buffer, pH 7.4). The brains were then removed, fixed overnight in paraformaldehyde, hydrated in a series of ethanol concentrations, and then paraffin-embedded. The brains were then cut into 5 µm sections using a paraffin slicing machine (Themo Fisher scientific, Waltham, MA, United States). The sections were dewaxed and dehydrated, and then antigen retrieval was performed by immersing the sections in sodium citrate buffer (10 mM, pH 6.0) for 5 min in a boiling water bath. Brain tissue sections were then blocked with 5% bovine serum albumin (BSA, Beyotime, Shanghai, China) at room temperature for 30 min. Then, the sections were incubated with anti-CD31 (1: 500 dilution) antibody at 4°C overnight. The next day, the sections were washed with PBST and incubated with fluorochrome-conjugated secondary antibody (1:500 dilution) at room temperature for 1 h. Finally, the sections were stained with DAPI (Beyotime, Shanghai, China) and visualized under a Nikon ECLPSE 80i fluorescence microscope (Nikon, Tokyo, Japan).

### Immunohistochemical Staining

Brain sections were deparaffinized, hydrated, incubated with 3% hydrogen peroxide for 10 min, and then boiled in sodium citrate buffer for 5 min. Brain tissue sections were then blocked with 5% BSA at room temperature for 30 min and then incubated with anti-MAP2 (1: 400 dilution) antibody at 4°C overnight. Subsequently, the sections were treated with biotinylated anti-mouse secondary antibody (1: 500 dilution) at room temperature for 1 h, stained with DAB reagent (ZSGB-BIO, Beijing, China) for 5 min, and counterstained with hematoxylin for 2 min. Finally, the sections were analyzed by light microscopy (Nikon, Tokyo, Japan).

### Nissl Staining

Nissl staining was used to investigate neuronal cell structure and neuronal damage. Brain sections were stained with Nissl staining solution (Beyotime, Shanghai, China) for 10 min in accordance with the manufacturer’s instructions. The density of neurons in the ipsilateral cortex was then determined by bright field microscopy (Nikon, Tokyo, Japan).

### Cell Culture

Primary human brain microvascular endothelial cells (HBMECs) were purchased from Cell Systems Corporation (ACBRI376, Kirkland, WA, United States). The cells were cultured in EBM-2 medium supplemented with 10% fetal bovine serum (FBS) and 1% penicillin-streptomycin in a humidified atmosphere at 37°C with 5% CO_2_.

### Oxygen-Glucose Deprivation/Reoxygenation and Drug Exposure

The oxygen and glucose deprivation-reoxygenation (OGD) model is an established *in vitro* ischemic model (Vijayan et al., 2019). In brief, after 72 h of high glucose (30 mM) treatment, HBMECs were treated with or without nmFGF1 (50, 100, 200 nM) in glucose and serum-free DMEM medium. The cells were then immediately placed in an anaerobic chamber (Themo scientific, MA, United States) saturated with mixed gas containing 95% N_2_ and 5% CO_2_ at 37°C for 4 h. After OGD exposure, the cells were maintained in EBM-2 with 10% FBS under normal conditions for 20 h.

### Cell Viability Assay

HBMEC cells were seeded into 96-well plates at a density of 1 × 10^4^ cells/well and treated with high glucose and OGD. The cells were then incubated with 200 µL of 0.5 mg/ml MTT solution at 37°C for 3 h. The supernatant was discarded and the formazan was dissolved with 150 µL DMSO. The optical density (*A* value) was then measured at a wavelength of 570 nm using a microplate reader (Bio Tek, Winooski, Vermont, United States). Cell viability was calculated using the following equation: cell viability (%) = [1–(*A*_control_–*A*_sample_)/(*A*_control_–*A*_blank_)] × 100%.

### Tube Formation Assay

The tube formation assay was used to evaluate *in vitro* angiogenesis. HBMEC cells were digested with 0.25% trypsin after treatment with HG + OGD alone or in combination with nmFGF1 (50, 100 and 200 nM), compound C (AMPK inhibitor, 5 µM), or A-769662 (AMPK agonist, 10 nM). The treated cells were then seeded into Matrigel-coated plates (Corning, Ithaca, NY, United States) at a density of 2 × 10^4^ cells/well, and cultured in EBM-2 medium at 37°C for 16 h. Tube formation was then observed by phase contrast microscopy (Leica, Oskar, Germany). The number of tubes was counted in three wells from each group.

### Wound Healing Assay

The wound healing assay is an effective method with which to assess cell migration (Li S. et al., 2020; Zhang et al., 2020). After OGD treatment, the cell monolayers were scratched off with 10 μL micropipette tips. The wells were then rinsed with PBS and incubated in EBM-2 medium supplemented with 1% FBS for 24 h. Cells that had migrated onto the scratched-off region were photographed under a microscope (Leica, Oskar, Germany) and the scratched gaps were quantified using ImageJ software (NIH, Bethesda, Maryland, United States). Cell migration was calculated using the following equation: migration ratio (%) = [(pre-migratory scratched open area–post-migratory open area)/(pre-migratory scratched open area)] × 100%.

### SiRNA Transfection

Human siRNA targeting AMPK (si-AMPK), and a negative control (si-con), were purchased from GenePharma (Shanghai, China). The sequences of the si-AMPK duplex were as follows: sense strand, 5′-GGA​CAG​GGA​AGC​CUU​AAA​UTT-3´; antisense strand, 5′-AUU​UAA​GGC​UUC​CCU​GUC​CTT-3´. The sequences of the negative control siRNA duplex were as follows: sense strand, 5′-UUC UCC​GAA​CGU​GUC​ACG​UTT-3´; antisense strand, 5′-ACG​UGA​CAC​GUU​CGG​AGA​ATT-3´. HBMEC cells were then transfected with 20 nM si-AMPK or si-con using Lipofectamine^®^ 2000 (Invitrogen, Carlsbad, CA, United States) in accordance with the manufacturer’s instructions. The transfected cells were used for subsequent experiments after they had been cultured for 48 h.

### Primary Neuron Culture and Condition Medium Testing

Primary cerebral cortex neurons were isolated from rat embryos at 16–18 days of gestation. The cerebral cortex was dissected, shredded, trypsinized (0.25%), and dissociated with a pipette. The lysates were then centrifuged, suspended, and filtered with a 40 µm nylon mesh (Corning, Ithaca, NY, United States). Cells were cultured on poly-d-lysine-coated 6 well plates at a density of 1 × 10^6^ cells/well. On day 7–9 of primary neuronal culture, neurons were treated with high glucose (30 mM) for 72 h, exposed to OGD for 30 min followed by reoxygenation for 24 h in the medium. The medium was prepared from fresh neuronal medium mixed with different treatment of conditioned medium (1: 4). Conditioned medium from replacement medium after HG + OGD-cultured HBMECs treatment with or without nmFGF1 for 24 h, the former referred to as CM and the latter as Veh. In addition, the conditioned medium from compound C-treated HBMEC cells cultured in CM, we referred to as CM-CC.

### Statistical Analysis

All data were derived from at least three independent experiments. Data are presented as the mean ± SEM and were analyzed by one-way analysis of variance (ANOVA) followed by Tukey’s post-hoc test. For the Morris water maze task, we use two-way analysis of variance followed by Tukey’s post-hoc test. Statistical analysis was carried out with Statistics Package for Social Science (SPSS) software (version 17.0; SPSS, IL, United States). *p* < 0.05 was considered to be statistically significant.

## Results

### The Combination of STZ and a HFD Successfully Induced a Model of Type 2 Diabetes

**FIGURE 1:**
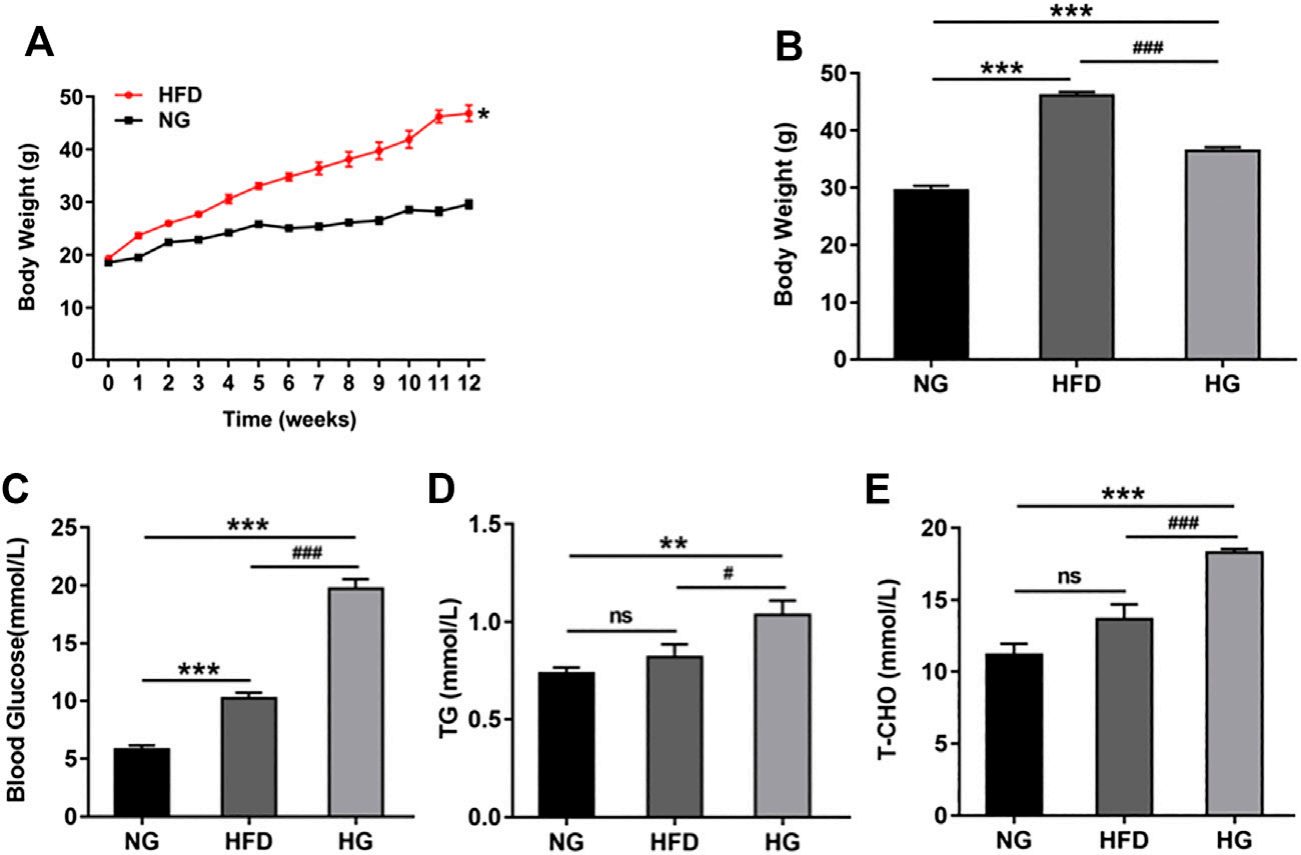
A type 2 diabetic model was established by a high-fat diet (HFD) and STZ injection **(A)** The changes of body weight in mice during the process of HFD (n = 10). **(B)** Body weight before and after STZ injection (n = 10). **(C–E)** The effects of HFD-STZ on the levels of fasting blood glucose, serum triglycerides, and total cholesterol, in mice (n = 6). Data are presented as means ± SEM. ^*^ *p* < 0.05, **p < 0.01, ***p < 0.001 vs. normal glucose (NG) group; ^#^ *p* < 0.05, ^###^ *p* < 0.001 vs. HFD group.

A mouse model of type 2 diabetes can be induced by a HFD and a low dose of STZ (Liu et al., 2019; Maremanda et al., 2020). In the present study, mice were fed with a HFD for 12 weeks prior to the injection of STZ. After 6 weeks, the HFD-fed mice showed a dramatic increase in body weight when compared to the mice fed a normal diet (NG) (Figure 1A). By week 12, the HFD-fed mice had gained more weight than the mice fed a normal diet and exhibited typical characteristics of obesity. However, after STZ injection, the body weight showed a significant reduction in the second week (Figure 1B). The levels of fasting blood glucose, triglyceride, and total cholesterol increased in mice fed HFD, and were markedly up-regulated (Figures 1C–E) in the mouse model of type 2 diabetes (HG). Collectively, these results indicated that we had successfully established a mouse model of type 2 diabetes by combining a HFD with a low dose of STZ.

### NmFGF1 Attenuated Infarct Size and Neurobehavioral Deficits in a Mouse Model of Diabetic Stroke

**FIGURE 2:**
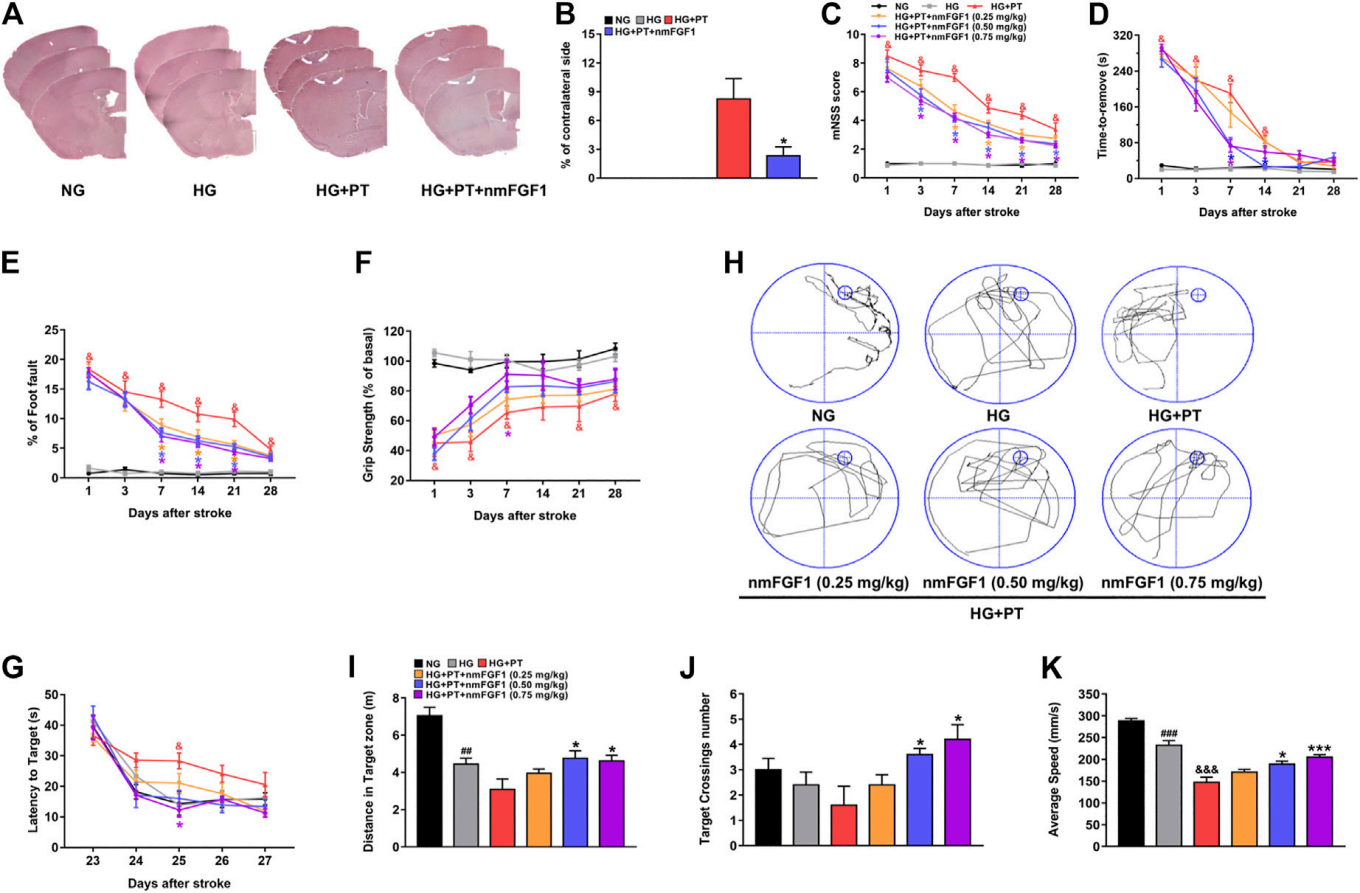
NmFGF1 attenuated infarct size and neurobehavioral deficits in a mouse model of diabetic stroke. **(A, B)** The infarction size on day 7 after ischemic stroke was observed by HE staining, and quantified by measuring the size of the cerebral infarction (n = 3). **(C–F)** Data derived from the mNSS, adhesive removal test, foot fault test, and grip strength test, on days 1, 3, 7, 14, 21, and 28 after stroke in diabetic mice (n = 8). Learning and memory deficits at 23–28 days after stroke were evaluated by the Morris water maze test. **(G)** Changes in the latency to reach the platform during the training period. **(H)** Representative images of swim routes to locate the platform in the mice in the different groups. **(I)** The distance traveled in the target quadrant. **(J)** The number of platform crossings during the probe trail. **(K)** Swimming speed in the platform quadrant (n = 5). Data are presented as means ± SEM. ^##^ *p* < 0.01, ^###^ *p* < 0.001 vs. NG group; ^&^ *p* < 0.05, ^&&&^ *p* < 0.001 vs. HG group; ^*^ *p* < 0.05, ^***^ *p* < 0.001 vs. HG + PT group.

Seven days after nmFGF1 administration, we collected and sectioned brain tissues for HE staining so that we could quantify the size of the lesions. The photothrombosis (HG + PT) induced diabetic stroke mice had a larger infarct (7.85% of contralateral side), and nmFGF1 significantly reduced infarct size of diabetic stroke mice (2.51% of contralateral side) (Figures 2A,B).

Previous authors have reported damage to the sensorimotor function of diabetic mice after stroke (Sharma et al., 2010). Therefore, in the present study, behavioral tests were performed on days 1, 3, 7, 14, 21, and 28, after stroke to investigate whether nmFGF1 improves the impairment of sensorimotor function in diabetic mice after stroke. The mNSS and adhesive removal test were used to evaluate somatosensory and neurological outcomes while the foot fault test and grip strength test were used to examine motor coordination ability. There were no significant differences between the NG group and the HG group in any of the behavioral tests. However, different doses of nmFGF1 attenuated the PT-induced neurological deficits in diabetic mice, particularly during the stroke recovery period (7–28 days) (Figures 2C–F). However, nmFGF1 treatment had no influence on the grip strength test except for on day 7 (Figure 2F).

Photochemical thrombosis is known to induce spatial learning and memory impairment in the frontal cortex by increasing oxidative stress (Rogers and Hunter, 1997; Song et al., 2012). We used the Morris water maze test to detect the learning and memory abilities of mice following the induction of lesions in the frontal cortex (Tucker et al., 2018). In all groups, escape latencies decreased with training (*F* (4, 96) = 58.55, *p* < 0.001) and escape latencies were significantly different among these groups (*F* (5, 24) = 7.121, *p* = 0.003). HG + PT mice required a longer time period to find the platform compared to the HG group; nmFGF1 treatment reduced the escape latencies (Figure 2G). In the probe test, the distance, frequency, and swimming speed, of platform crossing in the target quadrant were lower in the mice experiencing diabetic stroke; treatment with nmFGF1 (0.5, 0.75 mg/kg) increased the distance, frequency, and swimming speed, of platform crossings in the target quadrant (Figures 2H–K). These results revealed that nmFGF1 improved neurobehavioral impairment in diabetic ischemic stroke. Based on these data, nmFGF1 (0.25 mg/kg) did not significantly ameliorate neurobehavioral deficits in diabetic ischemic stroke. Moreover, there was no obvious change in the therapeutic effect of nmFGF1 at doses of 0.5 mg/kg to 0.75 mg/kg. Therefore, 0.5 mg/kg nmFGF1 was selected for subsequent *in vivo* experimental studies.

### NmFGF1 Promoted Angiogenesis in Diabetic Mice Following Ischemic Stroke

**FIGURE 3:**
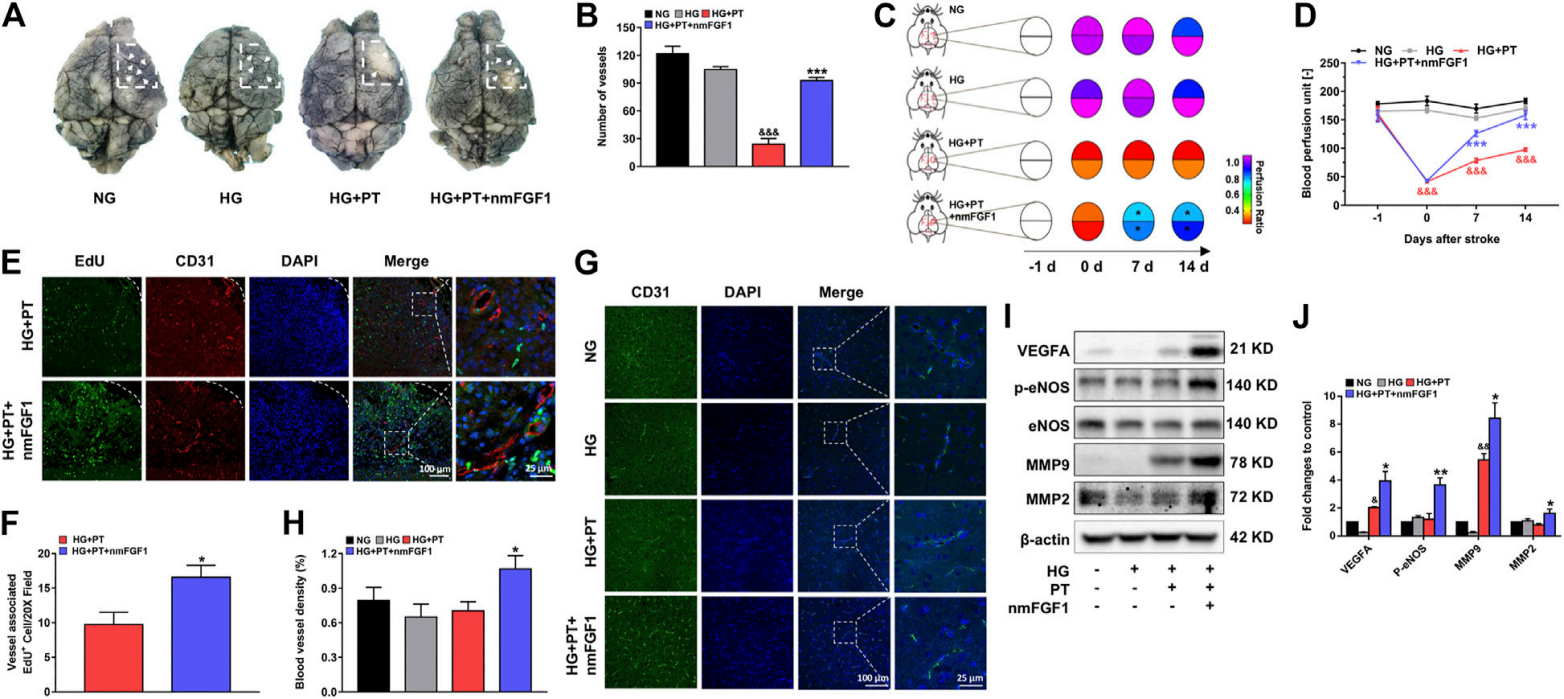
NmFGF1 promoted angiogenesis in a mouse model of diabetic stroke. **(A, B)** The cerebrovascular density within the ischemic zone on day 7 after ischemic stroke was examined by ink-gelatin perfusion and the quantification of cerebral vascular density (n = 3). **(C, D)** Blood perfusion in the ischemic area was evaluated by a laser doppler flowmeter and described by heat maps. Blood perfusion was quantified (n = 7). **(E)** Representative immunofluorescence images of DAPI and EdU staining along with staining of the vascular endothelium marker CD31 on day 7 after ischemic stroke. **(F)** Quantitative analysis of EdU^+^/CD31^+^ co-labeled cells in the ischemic area following stroke (n = 3). **(G)** The expression of CD31 7 days post-stroke as determined by immunofluorescence analysis. **(H)** Quantitative analysis of the intensity of CD31 (n = 3) **(I)** The expression levels of VEGFA, *p*-eNOS, eNOS, MMP9, and MMP2, as determined by western blot analysis. β-actin was used as a loading control **(J)** The band density of specific proteins was quantified by image software (n = 3). Data are presented as means ± SEM. ^&^ *p* < 0.05, ^&&^ *p* < 0.01, ^&&&^ *p* < 0.001 *vs.* HG group; ^*^ *p* < 0.05, **p < 0.01, ***p < 0.001 *vs.* HG + PT group.

It has been reported that FGF1 plays an important role in angiogenesis (Chen et al., 2020). Therefore, we investigated whether the therapeutic effect of nmFGF1 on diabetic stroke is related to the promotion of angiogenesis. Visualization of surface blood vessels showed that density of capillaries in diabetic mice was slightly lower than that of mice with normal glucose levels and was further aggravated by photochemical thrombosis. However, nmFGF1 treatment significantly improved the vascular density and integrity of the cerebral cortex (Figures 3A,B). These findings were supported by doppler measurements of cerebral blood flow (Figures 3C,D). Furthermore, we investigated the role of nmFGF1 to promote new angiogenesis on day 7 after stroke using EdU and CD31 immunofluorescent co-localization. CD31 is an endothelial marker that is indicative of angiogenesis. EdU was injected intraperitoneally to label proliferating cells. We found that when compared to the HG + PT group, nmFGF1 promoted new angiogenesis within the infarct area in diabetic stroke mice (Figures 3E,F).

In addition, the vascular density in the peri-infarcted area of brain tissues was detected by CD31 immunofluorescent staining. We found that nmFGF1 significantly increased the intensity of CD31 fluorescence in the ischemic brain tissue of the diabetic stroke group; there were no significant differences among the other groups (Figures 3G,H). Furthermore, we examined the expression levels of proteins related to angiogenesis by immunoblotting. The expression levels of VEGF, *p*-eNOS, MMP9, and MMP2, were all up-regulated in nmFGF1-treated mice experiencing diabetic stroke (Figures 3I,J). It is worth noting that MMP9 and MMP2 promote the migration of vascular endothelial cells by degrading the basement membrane (Zhang et al., 2019) and that endothelial cell migration plays an essential role in angiogenesis (Seto et al., 2016). Therefore, these results suggested that angiogenesis, mediated by the migration of endothelial cells, might play a positive role in the functional recovery of nmFGF1 after diabetic stroke injury**.**

### NmFGF1 Promoted Tube Formation and Migration in HG + OGD-Cultured HBMEC Cells

**FIGURE 4:**
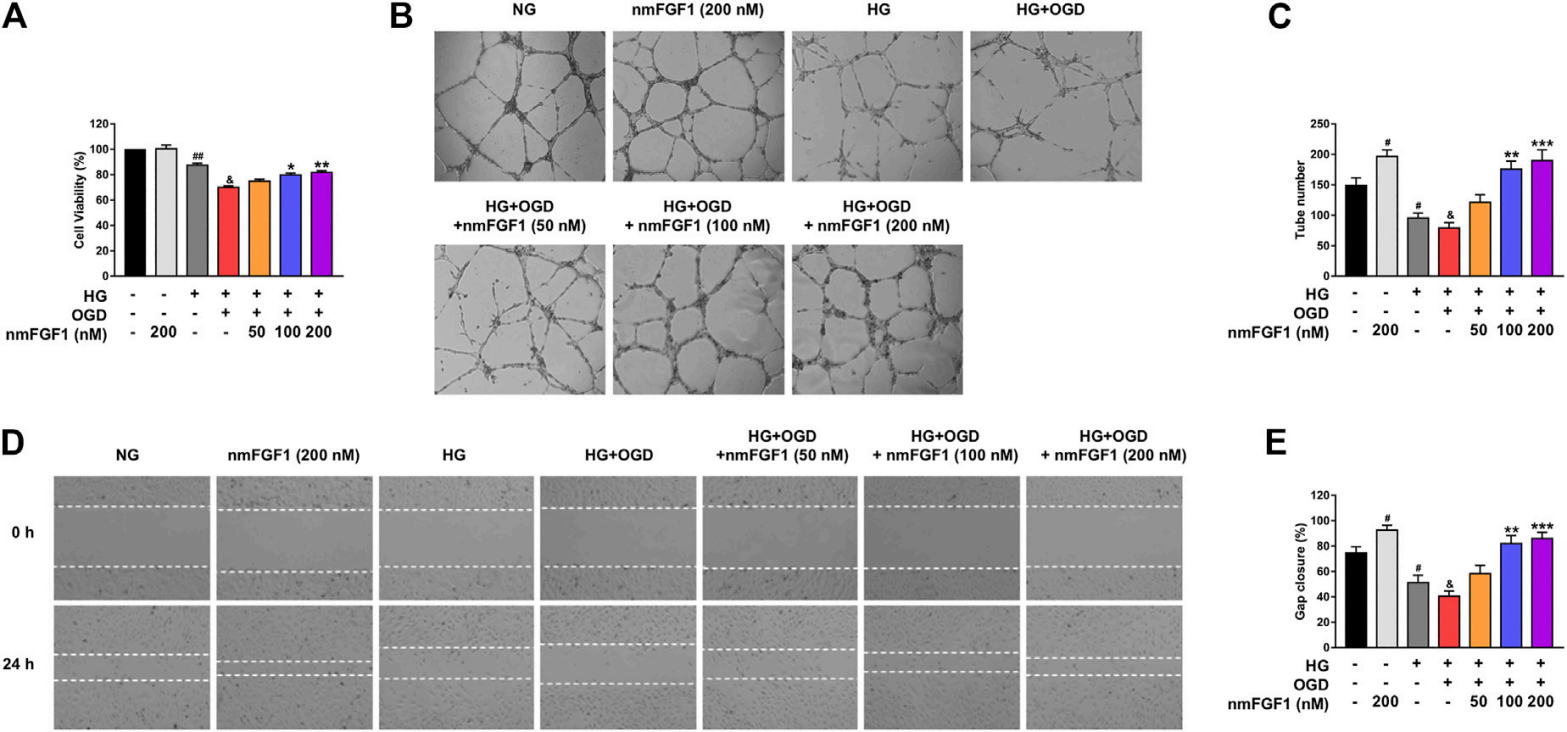
NmFGF1 reversed the reduction of tube formation and cell migration in HG + OGD-treated HBMEC cells. **(A)** The effect of nmFGF1 on the viability of HG + OGD-treated HBMEC cells. **(B)** The effects of nmFGF1 on tube formation, original magnification: ×40. **(C)** Quantitative analysis of the number of capillary-like tubes. **(D)** Wounding healing migration assay of HBMEC cells; images show wound areas as observed by phase-contrast microscopy, original magnification: ×40. **(E)** The migration ratio was calculated using Image Pro Plus software (n = 3). Date are presented as means ± SEM. ^#^ *p* < 0.05, ^##^ *p* < 0.01 vs. NG group; ^&^ *p* < 0.05 vs. HG group; ^*^ *p* < 0.05, **p < 0.01, ***p < 0.001 vs. HG + OGD group.

Next, we examined the effect of nmFGF1 on the cell viability of HBMEC cells cultured in high glucose conditions in combination with oxygen-glucose deprivation. As shown in Figure 4A, we found that nmFGF1 (100, 200 nM) reversed the pro-death effect of oxygen-glucose deprivation on HBMEC cells cultured in high glucose conditions. Moreover, nmFGF1 (200 nM) did not influence the proliferation of HBMEC cells when cultured in normal conditions. These results suggested that the protective effect of nmFGF1 on HBMEC cells may not be related to the promotion of cell proliferation.

Next, we investigated whether nmFGF1-mediated angiogenesis was involved in the functional improvement of diabetic stroke mice. Matrigel tube formation was performed as an *in vitro* experiment of angiogenesis to examine the effect of nmFGF1 on angiogenesis in HG + OGD-treated HBMEC cells. The treatment of HBMEC cells with HG + OGD markedly reduced the number of tubular structures compared with the HG-treated group. However, nmFGF1 reversed HG + OGD-impaired tube formation capacity in a dose-dependent manner while nmFGF1 (100, 200 nM) significantly increased the numbers of tubes formed when impaired by HG + OGD treatment (Figures 4B,C). These results indicated that nmFGF1 promoted angiogenesis in HG + OGD-treated HBMEC cells.

Next, we verified the effects of nmFGF1 on endothelial cell migration by the application of scratch wound healing assays. HG + OGD treatment suppressed cell migration while nmFGF1 (100, 200 nM) significantly reversed this inhibition. Moreover, 200 nM nmFGF1 significantly promoted cell migration under normal oxygen conditions (Figures 4D,E). These results further revealed that nmFGF1-induced endothelial cell migration may play a role in angiogenesis.

### NmFGF1 Promoted Tube Formation and Cell Migration by Activating the AMPK Signaling Pathway in HG + OGD-Treated HBMEC Cells

**FIGURE 5:**
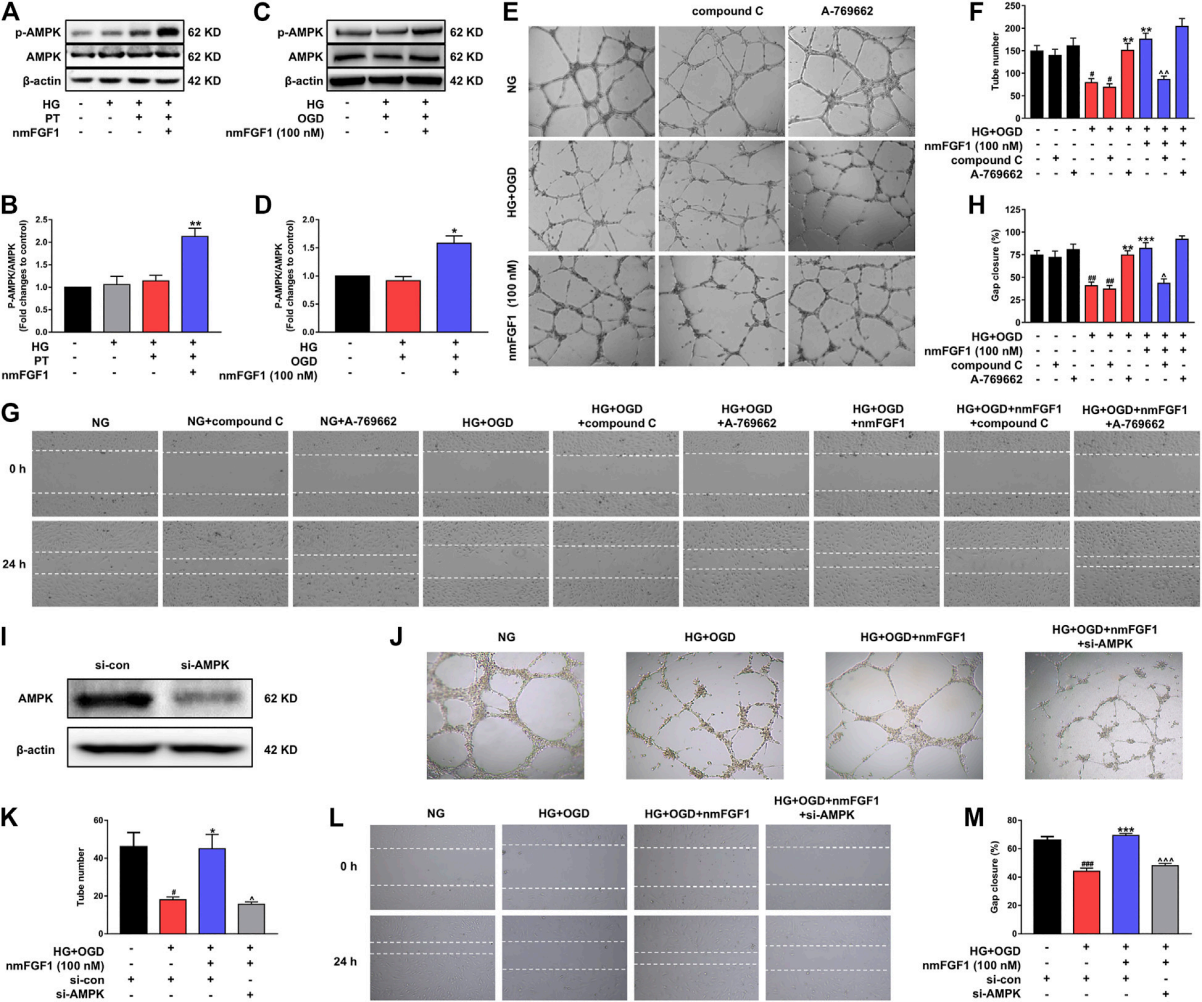
NmFGF1 promoted tube formation and migration of HBMEC in HG + OGD-treated HBMEC cells by activating the AMPK signaling pathway. **(A)** Western blotting analysis of *p*-AMPK and AMPK levels in a mouse model of diabetic stroke treated with or without nmFGF1. β-actin was used as a loading control. **(B)** The ratio of *p*-AMPK/AMPK. **(C)** The levels of *p*-AMPK and AMPK in HBMEC cells, as determined by western blotting. **(D)** The ratio of *p*-AMPK/AMPK in HBMECs, as quantified by image software. **(E)** Cells treated with an AMPK agonist or inhibitor. AMPK agonist, A-769662, 10 nM; AMPK inhibitor, compound C, 5 μM. Tube formation was observed by phase contrast microscopy, original magnification: ×40. **(F)** The number of tubes formed in HBMECs treated with an AMPK agonist or inhibitor. **(G)** The migration ability of HG + OGD-cultured HBMECs treated with an AMPK agonist or inhibitor. Images of the wound areas were observed by phase-contrast microscopy. A-769662, 10 nM, compound C, 5 μM; Original magnification: ×40. **(H)** The migration ratio was calculated using Image Pro Plus software. **(I)** AMPK expression was silenced by the transfection of siRNA. **(J)** The effect of AMPK silencing on tube formation. Original magnification: ×40. **(K)** Quantitative analysis of tube formation. **(L)** Wounding healing migration assays of cells in which AMPK had been silenced by siRNA. Original magnification: ×40. **(M)** Quantitative analysis of migration ratio (n = 3). Data are presented as means ± SEM. ^#^ *p* < 0.05, ^##^ *p* < 0.01, ^###^ *p* < 0.001 vs. NG group; ^*^ *p* < 0.05, **p < 0.01, ***p < 0.001 vs. HG + OGD or HG + PT group; ^^^ *p* < 0.05, ^^^^ *p* < 0.01, ^^^^^ *p* < 0.001 vs. HG + OGD + nmFGF1 group.

AMPK plays a critical role in the regulation of angiogenesis in endothelial cells (Avraham et al., 2013; Sowers et al., 2019). Therefore, we investigated whether the AMPK signaling pathway is involved in the angiogenesis mediated by nmFGF1. First, we detected the effect of nmFGF1 on the level of *p*-AMPK protein in the cerebral infarct cortex 7 days after stroke. NmFGF1 treatment significantly upregulated the expression of *p*-AMPK protein in diabetic stroke mice (Figures 5A,B). In addition, we also detected the expression of *p*-AMPK protein in HG + OGD-treated HBMEC cells after nmFGF1 treatment by immunoblotting. There were no significant differences in the levels of *p*-AMPK protein in HBMEC cells when compared between HG + OGD-treated and non-treated conditions. However, nmFGF1 significantly up-regulated the expression of *p*-AMPK protein in HG + OGD-treated HBMEC cells (Figures 5C,D). These results suggest that AMPK activation might be involved in the process of angiogenesis in nmFGF1-treated diabetic stroke mice. Therefore, to identify whether the AMPK signaling pathway was involved in the angiogenic process mediated by nmFGF1, we added an AMPK inhibitor compound C (5 µM), and AMPK agonist A-769662 (10 nM) to cell cultures. We found that the administration of compound C and A-769662 alone did not influence the tube formation ability of HBMEC cells when cultured in normal media. However, the HG + OGD-treated impairment of tube formation was significantly reversed by the administration of nmFGF1 or A-769662. However, compound C inhibited tube formation in the HG + OGD group with or without nmFGF1 treatment (Figures 5E,F). Next, we investigated whether nmFGF1 promoted tube formation in HBMEC cells *via* AMPK-mediated cell migration. We used compound C and A-769662 to treat HBMEC cells with or without nmFGF1 and found that HG + OGD-inhibited cell migration was reversed by A-769662 or nmFGF1 treatment, but this effect of nmFGF1 was suppressed by compound C treatment (Figures 5G,H).

We also transfected cells with AMPK-targeted siRNA to confirm that AMPK affects tube formation as well as cell migration (Figure 5I). We found that AMPK knockdown inhibited both tube formation and cell migration (Figure 5J–M). These results indicated that nmFGF1-promoted cell migration, mediated by the activation of the AMPK signaling pathway, played a key role on the angiogenesis of HBMECs under HG + OGD conditions.

### NmFGF1 Protected Ischemic Neurons by Promoting AMPK-Mediated Angiogenesis in a Mouse Model of Diabetic Stroke

**FIGURE 6:**
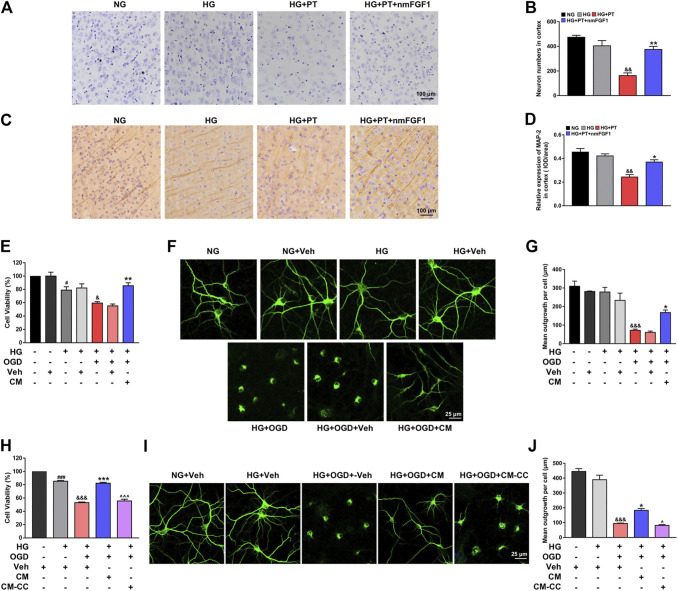
NmFGF1 ameliorated neuronal loss in a mouse model of diabetic stroke *via* AMPK-mediated angiogenesis. **(A)** Representative images of Nissl staining in the ipsilateral cortex region on day 7 after ischemic stroke. **(B)** The number of neurons in the cortex subfield were statistically analyzed with Image-Pro Plus software. **(C)** Representative images of immunohistochemical staining MAP-2 on day 7 after ischemic stroke. **(D)** Quantitative analysis of MAP-2 expression. Scale bar = 100 μm. **(E)** Effect of endothelial conditioned medium on the viability of HG + OGD-treated neurons. **(F, G)** Representative images of MAP2 immunofluorescence staining and quantitative analysis. **(H)** The effect of compound C treated endothelial conditioned medium on the viability of HG + OGD-treated neurons. **(I, J)** Representative images of MAP2 immunofluorescence staining and quantitative analysis. Scale bar = 25 μm. Conditioned medium from replacement medium after HG + OGD-cultured HBMECs treatment with or without nmFGF1 for 24 h, the former referred to as CM and the latter as Veh. In addition, the conditioned medium from compound C-treated HBMEC cells cultured in CM, we referred to as CM-CC. Data are presented as means ± SEM (n = 3). ^#^ *p* < 0.05, ^##^ *p* < 0.01 vs. NG or NG + Veh group; ^&^ *p* < 0.05, ^&&^ *p* < 0.01, ^&&&^ *p* < 0.001 vs. HG or HG + Veh group; ^*^ *p* < 0.05, **p < 0.01, ***p < 0.001 vs. HG + OGD or HG + OGD + Veh group. ^^^ *p* < 0.05, ^^^^^ *p* < 0.001 vs. HG + OGD + CM group.

NmFGF1 promoted functional recovery after cerebral ischemia in diabetic mice. We aimed to identify whether this functional recovery is associated with improvements to neuronal injury. To this end, we investigated the protective effect of nmFGF1 on neuronal damage after cerebral ischemia in diabetic mice. The density and morphology of neurons were observed with Nissl staining. We found that HG conditions led to a slight reduction in neuronal loss and injury to the neuronal structure and that these effects were further aggravated by PT treatment. However, treatment with nmFGF1 reduced neuronal loss and reversed injury to the neuronal structure (Figures 6A,B). Moreover, nmFGF1 significantly reversed the inhibition of MAP-2 expression by HG + PT in the cortex (Figures 6C,D).

Our results indicated that nmFGF1 promoted angiogenesis after stroke injury *via* the AMPK signaling pathway. This allowed the damaged brain to enter the repair period more quickly, thereby promoting functional recovery after cerebral ischemia in diabetic mice. However, whether the effects of nmFGF1 on the improvement of damaged neurons after cerebral ischemia is directly related to angiogenesis still needs to be verified. Therefore, first, we investigated the effects of HG + OGD-treated endothelial cells on neuronal viability and the role of nmFGF1 *in vitro*. MTT results showed that the conditioned medium from HG + OGD-treated HBMEC cells did not influence cell viability in HG + OGD-cultured neurons, while the conditioned medium from nmFGF1 significantly increased the survival rate of neurons (Figure 6E). MAP2 immunofluorescence further showed that nmFGF1-treated conditioned medium (CM) significantly improved the structure of neuronal protrusions in the HG + OGD-injured neurons (Figures 6F,G). The medium from endothelial cells treated with HG + OGD were used as negative controls for this experiment (Veh). The negative medium showed no difference in terms of cell survival and neurite structure when compared across all groups. These results indicated that HG + OGD-injured endothelial medium alone had no effect on neuronal survival and structure. Therefore, the neurons treated with the negative medium were directly used as a control group for subsequent studies.

Next, we investigated the effects of AMPK-mediated angiogenesis on neuronal protection. Therefore, we collected the conditioned medium from compound C-treated HBMEC cells cultured in CM (referred to as CM-CC) for the treatment of HG + OGD-cultured neurons. We found that CM-CC inhibited the protection of cell viability and morphological structure provided by CM treatment in the HG + OGD-cultured neurons (Figures 6H–J). These results suggested that nmFGF1 protected ischemic neurons by promoting AMPK-mediated angiogenesis, thereby enhancing functional recovery after cerebral ischemia in diabetic mice.

### NmFGF1 was Responsible for the Amelioration of Glucolipid Metabolism in a Mouse Model of Type 2 Diabetes After Stroke

**FIGURE 7:**
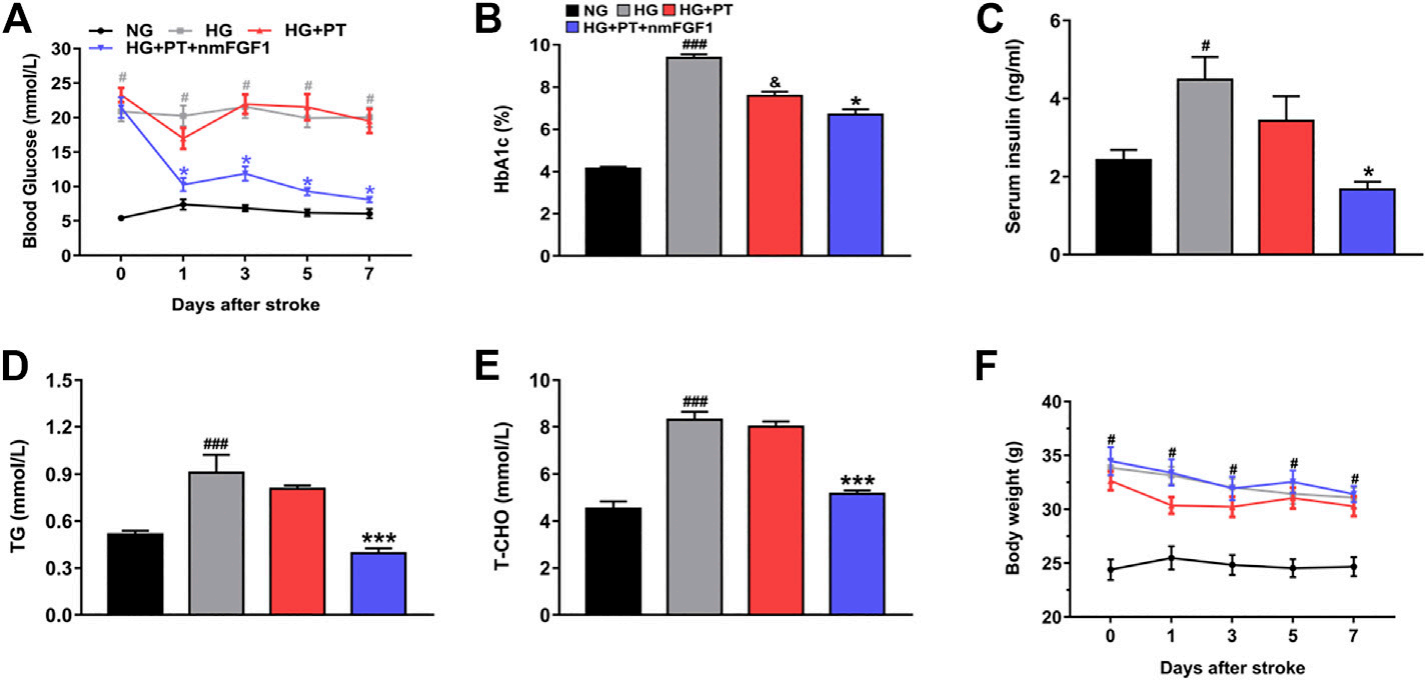
NmFGF1 ameliorated disorders of glucose and lipid metabolism in a mouse model of type 2 diabetic stroke. **(A)** Changes of blood glucose in diabetic mice were continuously monitored for 7 days after stroke (n = 8). **(B–E)** The effects of nmFGF1 on the levels of HbA1c, insulin, triglycerides, and total cholesterol, on day 7 after ischemic stroke (n = 6). **(F)** The changes of body weight in diabetic mice were continuously monitored for 7 days after stroke (n = 8). Data are presented as mean ± SEM. ^#^ *p* < 0.05, ^###^ *p* < 0.001 vs. NG group; ^&^ *p* < 0.05 vs. HG group; ^*^ *p* < 0.05, ***p < 0.001 vs. HG + PT group.

Hyperglycemia is known to aggravate neurological deficits and enlarge infarct size after stroke (Altinzo et al., 2015). Therefore, the precise regulation of glucolipid metabolism is necessary for the improvement of brain injury caused by diabetic stroke. We found that the levels of blood glucose, HbA1c, insulin and blood lipids, were significantly up-regulated in the HG-treated group when compared to the NG-treated group. However, treatment with nmFGF1 reduced the levels of blood glucose, HbA1c, insulin, triglycerides, and total cholesterol, in diabetic stroke mice (Figures 7A–E). In addition, we investigated whether nmFGF1-influenced body weight while improving glucolipid metabolism. The treatment of mice with STZ-HFD resulted in enhanced body weight compared with NG-treated mice. However, there was no significant difference in body weight when compared between diabetic stroke mice injected with nmFGF1 and mice that did not receive nmFGF1 (Figure 7F). These results indicated that nmFGF1 improved dysfunctional glucolipid metabolism but did not influence body weight in a mouse model of diabetic stroke.

## Discussion

Ischemic stroke is one of the most serious complications of diabetes and diabetic stroke is known to be associated with higher rates of mortality and disability. Therefore, the mechanisms and treatment of diabetic stroke have become a significant target for pharmaceutical researchers. In order to explore the mechanisms of cerebral ischemia and develop new drugs for stroke therapy, various animal models of ischemic stroke have been developed; these models feature middle cerebral artery occlusion (MCAO), craniotomy, and photothrombosis. MCAO is the most widely used model as it provides strict control and accuracy with regards to the timing of ischemia and reperfusion. The craniotomy model is established by directly occluding the cerebral blood vessels; the resultant pathological changes are similar to those associated with ischemic stroke in humans. However, the MCAO and craniotomy models have certain drawbacks that need to be considered. For example, these models both require complex operations; the infarct volume is difficult to control and will exert influence over the results and increase mortality. The photothrombotic ring model of stroke overcomes these shortcomings (Fluri et al., 2015). The location and size of the injury, along with the degree of photothrombosis, can be well controlled and reproduced efficiently. Meantime, the photothrombosis model is associated with low mortality rates, low levels of invasiveness, and a low risk of unwanted hemorrhage (Cotrina et al., 2017). This model induces damage to the vascular endothelial cells, the aggregation of platelets and microvascular occlusion by the excitation of a special dye that has accumulated in the microvessels, thus results in consistent infarction in the cortex but without causing hypothalamic damage. (Demyanenko et al., 2015; Uzdensky et al., 2017). Furthermore, diabetes is known to exacerbate neurological deficits and increases mortality. Therefore, in the present study, we used the photochemical thrombus model to reduce mortality in a mouse model of diabetic stroke.

One of the main characteristics of cerebral ischemia in diabetes is that the brain damage is aggravated by blood vessel injury. Angiogenesis and remodeling are the main repair processes following stroke and hold promise for post-stroke treatment by enhancing oxygen and nutrient supply to the affected tissue (Beck and Plate, 2009). The generation of blood vessels will stabilize brain perfusion. Furthermore, VEGF, and other pro-angiogenic factors from vessel components, might promote neurogenesis and reinforce axonal outgrowth. As a consequence, angiogenesis promotes neurological recovery and improves the prognosis of patients with stroke. (Wu et al., 2018; Sun et al., 2020). Therefore, angiogenesis is crucial to the treatment of brain injury after diabetic stroke. FGF1, a member of the fibroblast growth factor family, is known to alleviate the myocardial ischemia and cardiac insufficiency caused by coronary heart disease by promoting angiogenesis (Zhao et al., 2012; Zhang et al., 2016). Furthermore, it has been reported that FGF1 promotes skin wound healing by enhancing the formation of vessels and granulation tissue (Mellin et al., 1992). Based on this previous evidence, we investigated the effect of nmFGF1-mediated angiogenesis on neurological function in a mouse model of diabetic stroke. We found that nmFGF1 promoted angiogenesis after stroke in diabetic mice. Endothelial cell migration is also known to be necessary for angiogenesis (Mühleder et al., 2020; Wu et al., 2020). We found that nmFGF1 significantly reversed the migration and tube formation of HBMEC cells inhibited by HG + OGD treatment. These results suggested that angiogenesis is involved in the neural functional recovery of nmFGF1-treated mice experiencing diabetic stroke and that this process was mediated by endothelial cell migration.

It has been reported that the activation of AMPK plays a protective in ischemic stroke. It has been reported that the activation of AMPK plays a protective in ischemic stroke. AMPK, as a cellular energy sensor, can resist cerebral ischemic injury by switching on catabolic ATP-producing pathways and switching off anabolic ATP-consuming pathways (Marinangeli et al., 2016; Jiang et al., 2018). Palmatine exerts its neuroprotective effect *via* the activation of the AMPK/Nrf2 pathway (Tang et al., 2021). The AMPK pathway is also involved in the effects of tPA on neuronal apoptosis and mitophagy following stroke (Cai et al., 2021). AMPK also plays vital roles in regulating the growth and metabolism of eukaryotic cells and other cellular processes, including cell polarity, migration, and proliferation (Hu and Liu, 2016; Yu et al., 2016; Wang et al., 2017). Cilostazol was previously shown to induce cell proliferation and migration by activating the AMPK signaling pathway and that this process played a role in the formation of vascular tubes in HG-cultured huvec cells (Tseng et al., 2016). In the present study, we found that the administration of an AMPK inhibitor, compound C, and AMPK-target siRNA, resulted in the repression of tube formation and cell migration in HG + OGD-cultured HBMEC cells treated with nmFGF1; these findings concur with those of previous studies. It was previously reported that berberine facilitates angiogenesis in ischemic stroke by modulating microglial polarization *via* the AMPK signaling pathway (Zhu et al., 2019). Furthermore, rhFGF21 treatment promoted functional recovery in experimental stroke by modulating microglia/macrophage polarization (Wang et al., 2020). In light of these observations, we hypothesize that nmFGF1 promotes angiogenesis *via* AMPK-dependent microglial polarization.

Diabetes increases the risk of stroke by three-fold, exacerbates neurological deficits, and increases mortality (Huang et al., 2019; Mangin et al., 2019). The pathology of diabetic stroke is complex and heterogenous; this level of complexity is associated with the cellular dysfunction induced by hyperglycemia. Hyperglycemia has been shown to delay diabetic wound healing by inducing dysfunction in the vascular endothelial cells (Fan et al., 2021). Therefore, the improvement of glucolipid metabolism disorders is one of the most effective strategies for the treatment of diabetes and related complications. FGF1, a multi-pleiotropic metabolic regulator, is known to modulate blood glucose in metabolic diseases, including type 2 diabetes, non-alcoholic fatty liver, and obesity. Furthermore, FGF1 can act as an insulin sensitizer to effectively reduce hyperglycemia in diabetes without adverse reactions (Scarlett et al., 2016; Gasser et al., 2017; Tennant et al., 2019). In addition, chronic treatment with FGF1 increases insulin-dependent glucose uptake and suppresses hepatic glucose production to achieve insulin sensitization (Suh et al., 2014). In the present study, we found that nmFGF1 ameliorated the levels of blood glucose, HbA1c, insulin, triglycerides, and cholesterol in a mouse model of type 2 diabetic stroke without causing significant changes in body weight. In addition, nmFGF1 is a modified form of FGF1 with a good safety profile, low levels of mitogenic activity (Suh et al., 2014). These findings suggest that nmFGF1 can be widely used for the treatment of metabolic diseases.

## Conclusion

This study demonstrated the effects of nmFGF1 on a mouse model of diabetic stroke. Using this model, we found that nmFGF1 treatment improved glucolipid metabolism and promoted angiogenesis *via* the sAMPK signaling pathway. These effects contributed to functional recovery, at least in part.

## Data Availability

The original contributions presented in the study are included in the article/Supplementary Material, further inquiries can be directed to the corresponding authors.

## References

[B1] Altinzo E. Oner Z. Elbe H. Vardi N. (2015). Neuro-Protective Effects of Crocin on Brain and Cerebellum Tissues in Diabetic Rats. Afr. J. Trad. Compl. Alt. Med. 11, 33–39. 10.4314/ajtcam.v11i6.2

[B2] Avraham Y. Dayan M. Lassri V. Vorobiev L. Davidi N. Chernoguz D. (2013). Delayed Leptin Administration after Stroke Induces Neurogenesis and Angiogenesis. J. Neurosci. Res. 91, 187–195. 10.1002/jnr.23147 23152300

[B3] Banerjee S. Bentley P. Hamady M. Marley S. Davis J. Shlebak A. (2014). Intra-Arterial Immunoselected CD34+ Stem Cells for Acute Ischemic Stroke. Stem Cells Trans. Med. 3, 1322–1330. 10.5966/sctm.2013-0178 PMC4214837 25107583

[B4] Beck H. Plate K. H. (2009). Angiogenesis after Cerebral Ischemia. Acta Neuropathol. 117 (5), 481–496. 10.1007/s00401-009-0483-6 19142647

[B5] Begum G. Song S. Wang S. Zhao H. Bhuiyan M. I. H. Li E. (2018). Selective Knockout of Astrocytic Na+/H+ Exchanger Isoform 1 Reduces Astrogliosis, BBB Damage, Infarction, and Improves Neurological Function after Ischemic Stroke. Glia 66, 126–144. 10.1002/glia.23232 28925083 PMC5705024

[B6] Cai Y. Yang E. Yao X. Zhang X. Wang Q. Wang Y. (2021). FUNDC1-dependent Mitophagy Induced by tPA Protects Neurons against Cerebral Ischemia-Reperfusion Injury. Redox Biol. 38, 101792. 10.1016/j.redox.2020.101792 33212415 PMC7679257

[B7] Chen M. Bao L. Zhao M. Cao J. Zheng H. (2020). Progress in Research on the Role of FGF in the Formation and Treatment of Corneal Neovascularization. Front. Pharmacol. 11, 111. 10.3389/fphar.2020.00111 32158390 PMC7052042

[B8] Chen Z. Peng I.-C. Sun W. Su M.-I. Hsu P.-H. Fu Y. (2009). AMP-activated Protein Kinase Functionally Phosphorylates Endothelial Nitric Oxide Synthase Ser633. Circ. Res. 104, 496–505. 10.1161/CIRCRESAHA.108.187567 19131647 PMC2761102

[B9] Cheng X. Wang Z. Yang J. Ma M. Lu T. Xu G. (2011). Acidic Fibroblast Growth Factor Delivered Intranasally Induces Neurogenesis and Angiogenesis in Rats after Ischemic Stroke. Neurol. Res. 33, 675–680. 10.1179/1743132810Y.0000000004 21756545

[B10] Cotrina M. L. Lou N. Tome-Garcia J. Goldman J. Nedergaard M. (2017). Direct Comparison of Microglial Dynamics and Inflammatory Profile in Photothrombotic and Arterial Occlusion Evoked Stroke. Neuroscience 343, 483–494. 10.1016/j.neuroscience.2016.12.012 28003156 PMC5523105

[B11] Demyanenko S. V. Panchenko S. N. Uzdensky A. B. (2015). Expression of Neuronal and Signaling Proteins in Penumbra Around a Photothrombotic Infarction Core in Rat Cerebral Cortex. Biochem. Mosc. 80, 790–799. 10.1134/S0006297915060152 26531025

[B12] Diederich K. Schmidt A. Strecker J.-K. Schäbitz W.-R. Schilling M. Minnerup J. (2014). Cortical Photothrombotic Infarcts Impair the Recall of Previously Acquired Memories but Spare the Formation of New Ones. Stroke 45, 614–618. 10.1161/STROKEAHA.113.001907 24347420

[B13] Fan J. Liu H. Wang J. Zeng J. Tan Y. Wang Y. (2021). Procyanidin B2 Improves Endothelial Progenitor Cell Function and Promotes Wound Healing in Diabetic Mice *via* Activating Nrf2. J. Cel Mol Med 25, 652–665. 10.1111/jcmm.16111 PMC7812287 33215883

[B14] Fluri F. Schuhmann M. K. Kleinschnitz C. (2015). Animal Models of Ischemic Stroke and Their Application in Clinical Research. Drug Des Devel Ther. 9, 3445–3454. 10.2147/DDDT.S56071 PMC4494187 26170628

[B15] Gasser E. Moutos C. P. Downes M. Evans R. M. (2017). FGF1 - a New Weapon to Control Type 2 Diabetes Mellitus. Nat. Rev. Endocrinol. 13, 599–609. 10.1038/nrendo.2017.78 28664920 PMC5839646

[B16] Guo Y. Chen X. Li D. Liu H. Ding Y. Han R. (2018). PR-957 Mediates Neuroprotection by Inhibiting Th17 Differentiation and Modulating Cytokine Production in a Mouse Model of Ischaemic Stroke. Clin. Exp. Immunol. 193, 194–206. 10.1111/cei.13132 29603201 PMC6046491

[B17] Hasan M. R. Herz J. Hermann D. M. Doeppner T. R. (2013). Intravascular Perfusion of Carbon Black Ink Allows Reliable Visualization of Cerebral Vessels. JoVE 71, e4374. 10.3791/4374 PMC3582667 23328838

[B19] Hu M. Liu B. (2016). Resveratrol *via* Activation of LKB1-AMPK Signaling Suppresses Oxidative Stress to Prevent Endothelial Dysfunction in Diabetic Mice. Clin. Exp. Hypertens. 38, 381–387. 10.3109/10641963.2015.1131288 27149559

[B20] Huang W.-y. Wu G. Guo S.-x. Geng D.-y. Li J.-j. Yang K. (2019). Multi-parameters of Magnetic Resonance Imaging to Estimate Ischemia-Reperfusion Injury after Stroke in Hyperglycemic Rats. Sci. Rep. 9, 2852. 10.1038/s41598-019-39263-6 30814576 PMC6393533

[B21] Jackson L. Dong G. Althomali W. Sayed M. A. Eldahshan W. Baban B. (2020). Delayed Administration of Angiotensin II Type 2 Receptor (AT2R) Agonist Compound 21 Prevents the Development of Post-stroke Cognitive Impairment in Diabetes through the Modulation of Microglia Polarization. Transl. Stroke Res. 11, 762–775. 10.1007/s12975-019-00752-5 31792796 PMC7266715

[B22] Jiang S. Li T. Ji T. Yi W. Yang Z. Wang S. (2018). AMPK: Potential Therapeutic Target for Ischemic Stroke. Theranostics 8 (16), 4535–4551. 10.7150/thno.25674 30214637 PMC6134933

[B23] Jiang Y. Liu N. Han J. Li Y. Spencer P. Vodovoz S. J. (2020). Diabetes Mellitus/Poststroke Hyperglycemia: a Detrimental Factor for tPA Thrombolytic Stroke Therapy. Transl. Stroke Res. 12, 416–427. 10.1007/s12975-020-00872-3 33140258

[B24] Jin Q. Cheng J. Liu Y. Wu J. Wang X. Wei S. (2014). Improvement of Functional Recovery by Chronic Metformin Treatment Is Associated with Enhanced Alternative Activation of Microglia/macrophages and Increased Angiogenesis and Neurogenesis Following Experimental Stroke. Brain Behav. Immun. 40, 131–142. 10.1016/j.bbi.2014.03.003 24632338

[B25] Jouihan S. A. Zuloaga K. L. Zhang W. Shangraw R. E. Krasnow S. M. Marks D. L. (2013). Role of Soluble Epoxide Hydrolase in Exacerbation of Stroke by Streptozotocin-Induced Type 1 Diabetes Mellitus. J. Cereb. Blood Flow Metab. 33, 1650–1656. 10.1038/jcbfm.2013.130 23899929 PMC3790937

[B26] Li S. Sun X. Xu L. Sun R. Ma Z. Deng X. (2017). Baicalin Attenuates In Vivo and In Vitro Hyperglycemia-Exacerbated Ischemia/reperfusion Injury by Regulating Mitochondrial Function in a Manner Dependent on AMPK. Eur. J. Pharmacol. 815, 118–126. 10.1016/j.ejphar.2017.07.041 28743390

[B27] Li S. Yan G. Liu W. Li C. Wang X. (2020). Circ0106714 Inhibits Tumorigenesis of Colorectal Cancer by Sponging miR‐942‐5p and Releasing DLG2 *via* Hippo‐YAP Signaling. Mol. carcinogenesis 59, 1323–1342. 10.1002/mc.23259 33128289

[B28] Li Z. Xiao G. Lyu M. Wang Y. He S. Du H. (2020). Shuxuening Injection Facilitates Neurofunctional Recovery *via* Down-Regulation of G-CSF-Mediated Granulocyte Adhesion and Diapedesis Pathway in a Subacute Stroke Mouse Model. Biomed. Pharmacother. 127, 110213. 10.1016/j.biopha.2020.110213 32417690

[B29] Lin Q. Huang Z. Cai G. Fan X. Yan X. Liu Z. (2020). Activating AMP‐activated Protein Kinase Mediates Fibroblast Growth Factor 1 Protection from Nonalcoholic Fatty Liver Disease in Mice. Hepatology. 10.1002/hep.31568 PMC8082952 32965675

[B30] Liu H. Zhao Y. Zou Y. Huang W. Zhu L. Liu F. (2019). Heparin‐poloxamer Hydrogel‐encapsulated rhFGF21 Enhances Wound Healing in Diabetic Mice. FASEB j. 33, 9858–9870. 10.1096/fj.201802600RR 31166803

[B31] Lu Q. Li X. Liu J. Sun X. Rousselle T. Ren D. (2019). AMPK Is Associated with the Beneficial Effects of Antidiabetic Agents on Cardiovascular Diseases. Biosci. Rep. 39, 1–15. 10.1042/bsr20181995 PMC6379227 30710062

[B32] Mangin G. Poittevin M. Charriaut-Marlangue C. Giannesini C. Merkoulova-Rainon T. Kubis N. (2019). Glatiramer Acetate Reduces Infarct Volume in Diabetic Mice with Cerebral Ischemia and Prevents Long-Term Memory Loss. Brain Behav. Immun. 80, 315–327. 10.1016/j.bbi.2019.04.009 30953775

[B33] Maremanda K. P. Srivalliputturu S. B. Jena G. (2020). Zinc Deficient Diet Exacerbates the Testicular and Epididymal Damage in Type 2 Diabetic Rat: Studies on Oxidative Stress-Related Mechanisms. Reprod. Biol. 20, 191–201. 10.1016/j.repbio.2020.03.002 32245730

[B34] Marinangeli C. Didier S. Vingtdeux V. (2016). AMPK in Neurodegenerative Diseases: Implications and Therapeutic Perspectives. Cdt 17 (8), 890–907. 10.2174/1389450117666160201105645 26073858

[B35] Mellin T. N. Mennie R. J. Cashen D. E. Ronan J. J. Capparella J. James M. L. (1992). Acidic Fibroblast Growth Factor Accelerates Dermal Wound Healing. Growth factors 7, 1–14. 10.3109/08977199209023933 1380253

[B36] Mühleder S. Fernández-Chacón M. Garcia-Gonzalez I. Benedito R. (2020). Endothelial Sprouting, Proliferation, or Senescence: Tipping the Balance from Physiology to Pathology. Cell. Mol. Life Sci. 78, 1329–1354. 10.1007/s00018-020-03664-y 33078209 PMC7904752

[B37] Rogers D. C. Hunter A. J. (1997). Photothrombotic Lesions of the Rat Cortex Impair Acquisition of the Water Maze. Pharmacol. Biochem. Behav. 56, 747–754. 10.1016/s0091-3057(96)00430-3 9130302

[B38] Rust R. Grönnert L. Gantner C. Enzler A. Mulders G. Weber R. Z. (2019). Nogo-A Targeted Therapy Promotes Vascular Repair and Functional Recovery Following Stroke. Proc. Natl. Acad. Sci. USA 116, 14270–14279. 10.1073/pnas.1905309116 31235580 PMC6628809

[B40] Scarlett J. M. RojasMatsen J. M. M. E. Matsen M. E. Kaiyala K. J. Stefanovski D. Bergman R. N. (2016). Central Injection of Fibroblast Growth Factor 1 Induces Sustained Remission of Diabetic Hyperglycemia in Rodents. Nat. Med. 22, 800–806. 10.1038/nm.4101 27213816 PMC4938755

[B41] Seto S.-W. Chang D. Jenkins A. Bensoussan A. Kiat H. (2016). Angiogenesis in Ischemic Stroke and Angiogenic Effects of Chinese Herbal Medicine. Jcm 5, 56–26. 10.3390/jcm5060056 PMC4929411 27275837

[B42] Sharma H. S. Patnaik R. Sharma A. (2010). Diabetes Aggravates Nanoparticles Induced Breakdown of the Blood-Brain Barrier Permeability, Brain Edema Formation, Alterations in Cerebral Blood Flow and Neuronal Injury. An Experimental Study Using Physiological and Morphological Investigations in the Rat. J. Nanosci. Nanotech. 10, 7931–7945. 10.1166/jnn.2010.3616 21121280

[B43] Song M.-K. Seon H.-J. Kim I.-G. Han J.-Y. Choi I.-S. Lee S.-G. (2012). The Effect of Combined Therapy of Exercise and Nootropic Agent on Cognitive Function in Focal Cerebral Infarction Rat Model. Ann. Rehabil. Med. 36, 303–310. 10.5535/arm.2012.36.3.303 22837964 PMC3400868

[B44] Sowers J. R. Habibi J. Aroor A. R. Yang Y. Lastra G. Hill M. A. (2019). Epithelial Sodium Channels in Endothelial Cells Mediate Diet-Induced Endothelium Stiffness and Impaired Vascular Relaxation in Obese Female Mice. Metabolism 99, 57–66. 10.1016/j.metabol.2019.153946 31302199 PMC6901094

[B45] Suda S. Ueda M. Nito C. Nishiyama Y. Okubo S. Abe A. (2015). Valproic Acid Ameliorates Ischemic Brain Injury in Hyperglycemic Rats with Permanent Middle Cerebral Occlusion. Brain Res. 1606, 1–8. 10.1016/j.brainres.2015.02.013 25721785

[B46] Suh J. M. Jonker J. W. Ahmadian M. Goetz R. Lackey D. Osborn O. (2014). Endocrinization of FGF1 Produces a Neomorphic and Potent Insulin Sensitizer. Nature 513, 436–439. 10.1038/nature13540 25043058 PMC4184286

[B47] Sun P. Zhang K. Hassan S. H. Zhang X. Tang X. Pu H. (2020). Endothelium-Targeted Deletion of microRNA-15a/16-1 Promotes Poststroke Angiogenesis and Improves Long-Term Neurological Recovery. Circ. Res. 126 (8), 1040–1057. 10.1161/CIRCRESAHA.119.315886 32131693 PMC7172020

[B48] Sutter S. Todorov A. Ismail T. Haumer A. Fulco I. Schulz G. (2017). Contrast-Enhanced Microtomographic Characterisation of Vessels in Native Bone and Engineered Vascularised Grafts Using Ink-Gelatin Perfusion and Phosphotungstic Acid. Contrast Media Mol. Imaging 2017, 1–10. 10.1155/2017/40351602017 PMC5612680 29097920

[B49] Tang C. Hong J. Hu C. Huang C. Gao J. Huang J. (2021). Palmatine Protects against Cerebral Ischemia/Reperfusion Injury by Activation of the AMPK/Nrf2 Pathway. Oxidative Med. Cell Longevity 2021, 1–12. 10.1155/2021/6660193 PMC7981182 33777318

[B50] Tennant K. G. Lindsley S. R. KirigitiTrue M. A. C. True C. Kievit P. (2019). Central and Peripheral Administration of Fibroblast Growth Factor 1 Improves Pancreatic Islet Insulin Secretion in Diabetic Mouse Models. Diabetes 68, 1462–1472. 10.2337/db18-1175 31048370 PMC6609981

[B51] Tseng S.-Y. Chao T.-H. Li Y.-H. Liu P.-Y. Lee C.-H. Cho C.-L. (2016). Cilostazol Improves High Glucose-Induced Impaired Angiogenesis in Human Endothelial Progenitor Cells and Vascular Endothelial Cells as Well as Enhances Vasculoangiogenesis in Hyperglycemic Mice Mediated by the Adenosine Monophosphate-Activated Protein Kinase Pathway. J. Vasc. Surg. 63, 1051–1062. 10.1016/j.jvs.2014.10.103 25595409

[B52] Tucker L. B. Velosky A. G. McCabe J. T. (2018). Applications of the Morris Water Maze in Translational Traumatic Brain Injury Research. Neurosci. Biobehavioral Rev. 88, 187–200. 10.1016/j.neubiorev.2018.03.010 29545166

[B53] Uzdensky A. Demyanenko S. Fedorenko G. Lapteva T. Fedorenko A. (2017). Protein Profile and Morphological Alterations in Penumbra after Focal Photothrombotic Infarction in the Rat Cerebral Cortex. Mol. Neurobiol. 54, 4172–4188. 10.1007/s12035-016-9964-5 27324898

[B54] Venna V. R. Li J. Hammond M. D. Mancini N. S. McCullough L. D. (2014). Chronic Metformin Treatment Improves Post-stroke Angiogenesis and Recovery after Experimental Stroke. Eur. J. Neurosci. 39, 2129–2138. 10.1111/ejn.12556 24649970 PMC4061245

[B55] Vijayan M. Alamri F. F. Al Shoyaib A. Karamyan V. T. Reddy P. H. (2019). Novel miRNA PC-5P-12969 in Ischemic Stroke. Mol. Neurobiol. 56, 6976–6985. 10.1007/s12035-019-1562-x 30953313

[B56] Wang D. Liu F. Zhu L. Lin P. Han F. Wang X. (2020). FGF21 Alleviates Neuroinflammation Following Ischemic Stroke by Modulating the Temporal and Spatial Dynamics of Microglia/macrophages. J. Neuroinflammation 17, 257. 10.1186/s12974-020-01921-2 32867781 PMC7457364

[B57] Wang D. Song Y. Zhang J. Pang W. Wang X. Zhu Y. (2017). AMPK-KLF2 Signaling Pathway Mediates the Proangiogenic Effect of Erythropoietin in Endothelial Colony-Forming Cells. Am. J. Physiology-Cell Physiol. 313, C674–C685. 10.1152/ajpcell.00257.2016 28978525

[B58] Wu C.-C. Wang L.-C. Su Y.-T. Wei W.-Y. Tsai K.-J. (2018). Synthetic α5β1 Integrin Ligand PHSRN Is Proangiogenic and Neuroprotective in Cerebral Ischemic Stroke. Biomaterials 185, 142–154. 10.1016/j.biomaterials.2018.09.014 30243150

[B59] Wu J. Zhu J. He C. Xiao Z. Ye J. Li Y. (2016). Comparative Study of Heparin-Poloxamer Hydrogel Modified bFGF and aFGF for In Vivo Wound Healing Efficiency. ACS Appl. Mater. Inter. 8, 18710–18721. 10.1021/acsami.6b06047 27384134

[B60] Wu X. Su Z. Li X. Zheng Q. Huang Y. Yuan H. (2005). High-level Expression and Purification of a Nonmitogenic Form of Human Acidic Fibroblast Growth Factor in *Escherichia coli* . Protein Expr. Purif. 42, 7–11. 10.1016/j.pep.2004.07.021 15882952

[B61] Wu X. Zhou J. Li D. (2020). Orientation of the Mitotic Spindle in Blood Vessel Development. Front. Cel Dev. Biol. 8, 583325. 10.3389/fcell.2020.583325 PMC7533553 33072763

[B62] Xue S. Gong H. Jiang T. Luo W. Meng Y. Liu Q. (2014). Indian-ink Perfusion Based Method for Reconstructing Continuous Vascular Networks in Whole Mouse Brain. PloS one 9, e88067. 10.1371/journal.pone.0088067 24498247 PMC3907580

[B63] Yasmeen S. Akram B. H. Hainsworth A. H. Kruuse C. (2019). Cyclic Nucleotide Phosphodiesterases (PDEs) and Endothelial Function in Ischaemic Stroke. A Review. Cell Signal. 61, 108–119. 10.1016/j.cellsig.2019.05.011 31132399

[B64] Yu J.-W. Deng Y.-P. Han X. Ren G.-F. Cai J. Jiang G.-J. (2016). Metformin Improves the Angiogenic Functions of Endothelial Progenitor Cells *via* Activating AMPK/eNOS Pathway in Diabetic Mice. Cardiovasc. Diabetol. 15, 88. 10.1186/s12933-016-0408-3 27316923 PMC4912824

[B65] Zhang M. Yu W.-Z. Shen X.-T. Xiang Q. Xu J. Yang J.-J. (2016). Advanced Interfere Treatment of Diabetic Cardiomyopathy Rats by aFGF-Loaded Heparin-Modified Microbubbles and UTMD Technique. Cardiovasc. Drugs Ther. 30, 247–261. 10.1007/s10557-016-6639-4 26947349

[B66] Zhang Q. Zhou M. Wu X. Li Z. Liu B. Gao W. (2019). Promoting Therapeutic Angiogenesis of Focal Cerebral Ischemia Using Thrombospondin-4 (TSP4) Gene-Modified Bone Marrow Stromal Cells (BMSCs) in a Rat Model. J. Transl Med. 17, 111. 10.1186/s12967-019-1845-z 30947736 PMC6449913

[B67] Zhang T. Jia W. Sun X. (2010). 3-n-Butylphthalide (NBP) Reduces Apoptosis and Enhances Vascular Endothelial Growth Factor (VEGF) Up-Regulation in Diabetic Rats. Neurol. Res. 32, 390–396. 10.1179/016164110X12670144526264 20483006

[B68] Zhang Z. Hou W. b. Zhang C. Tan Y. e. Zhang D. d. An W. (2020). A Research of STEAP1 Regulated Gastric Cancer Cell Proliferation, Migration and Invasion In Vitro and in Vivos. J. Cel. Mol. Med. 24, 14217–14230. 10.1111/jcmm.16038 PMC7754049 33128353

[B69] Zhao Y.-Z. Lu C.-T. Li X.-K. Tang Q.-Q. Tian X.-Q. Zhao Y.-P. (2012). Improving the Cardio Protective Effect of aFGF in Ischemic Myocardium with Ultrasound-Mediated Cavitation of Heparin Modified Microbubbles: Preliminary Experiment. J. Drug Target. 20, 623–631. 10.3109/1061186X.2012.702771 22758395

[B70] Zhu J. Cao D. Guo C. Liu M. Tao Y. Zhou J. (2019). Berberine Facilitates Angiogenesis against Ischemic Stroke through Modulating Microglial Polarization *via* AMPK Signaling. Cell Mol Neurobiol 39, 751–768. 10.1007/s10571-019-00675-7 31020571 PMC11462843

[B71] Zou Y. Hu J. Huang W. Ye S. Han F. Du J. (2020). Non-Mitogenic Fibroblast Growth Factor 1 Enhanced Angiogenesis Following Ischemic Stroke by Regulating the Sphingosine-1-Phosphate 1 Pathway. Front. Pharmacol. 11, 59. 10.3389/fphar.2020.00059 32194396 PMC7063943

