# Corrigendum: NmFGF1-regulated glucolipid metabolism and angiogenesis improves functional recovery in a mouse model of diabetic stroke and acts via the AMPK signaling pathway

**DOI:** 10.3389/fphar.2024.1392148

**Published:** 2024-05-17

**Authors:** Yeli Zhao, Shasha Ye, Jingjing Lin, Fei Liang, Jun Chen, Jian Hu, Kun Chen, Yani Fang, Xiongjian Chen, Ye Xiong, Li Lin, Xianxi Tan

**Affiliations:** ^1^ The First Affiliated Hospital of Wenzhou Medical University, Wenzhou, China; ^2^ School of Pharmaceutical Sciences, Wenzhou Medical University, Wenzhou, China; ^3^ Research Units of Clinical Translation of Cell Growth Factors and Diseases Research, Chinese Academy of Medical Science, Wenzhou, China

**Keywords:** ischemic stroke, diabetes, nmFGF1, angiogenesis, AMPK

In the published article, there was an error in Figure 4 and Figure 5 as published. The representative images and quantified data in Figures 4B, C and Figures 5G, H have been presented incorrectly. Two pictures were taken from the same cell culture well with HG treatment in Figure 4B and HG + OGD + nmFGF1+A-769662 treatment in Figure 5G, respectively. Therefore, we reassembled and quantified all pictures in Figures 4B, C and Figures 5G, H. The corrected Figure 4 and Figure 5 and its captions appear below.

**FIGURE 4:**
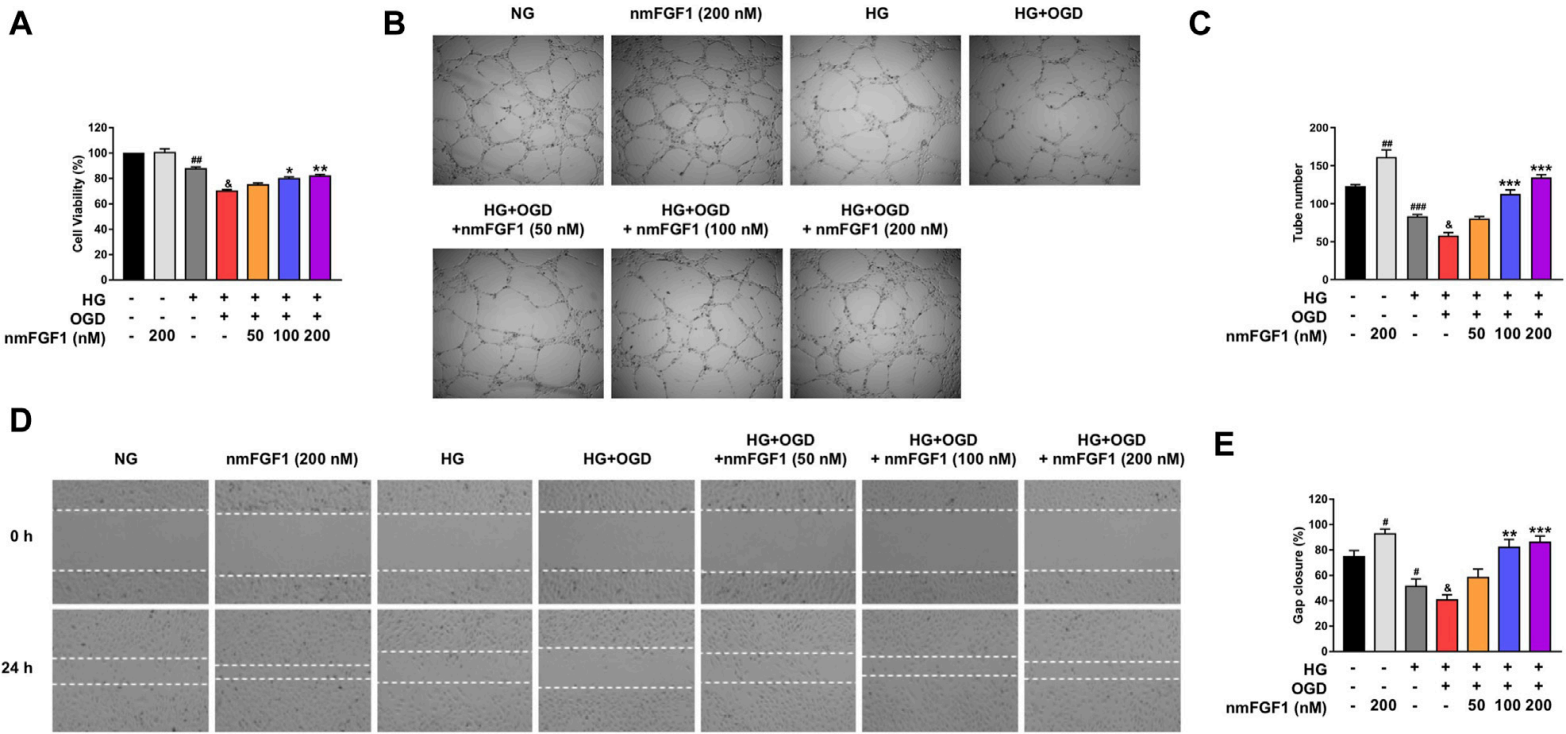
NmFGF1 reversed the reduction of tube formation and cell migration in HG + OGD-treated HBMEC cells. **(A)** The effect of nmFGF1 on the viability of HG + OGD-treated HBMEC cells. **(B)** The effects of nmFGF1 on tube formation, original magnification: ×40. **(C)** Quantitative analysis of the number of capillary-like tubes. **(D)** Wounding healing migration assay of HBMEC cells; images show wound areas as observed by phase-contrast microscopy, original magnification: ×40. **(E)** The migration ratio was calculated using Image Pro Plus software (*n* = 3). Date are presented as means ± SEM. ^#^ *p* < 0.05, ^##^ *p* < 0.01, ^###^ *p* < 0.001 vs. NG group; ^&^ *p* < 0.05 vs. HG group; ^*^ *p* < 0.05, ^**^ *p* < 0.01, ^***^ *p* < 0.001 vs. HG + OGD group.

**FIGURE 5:**
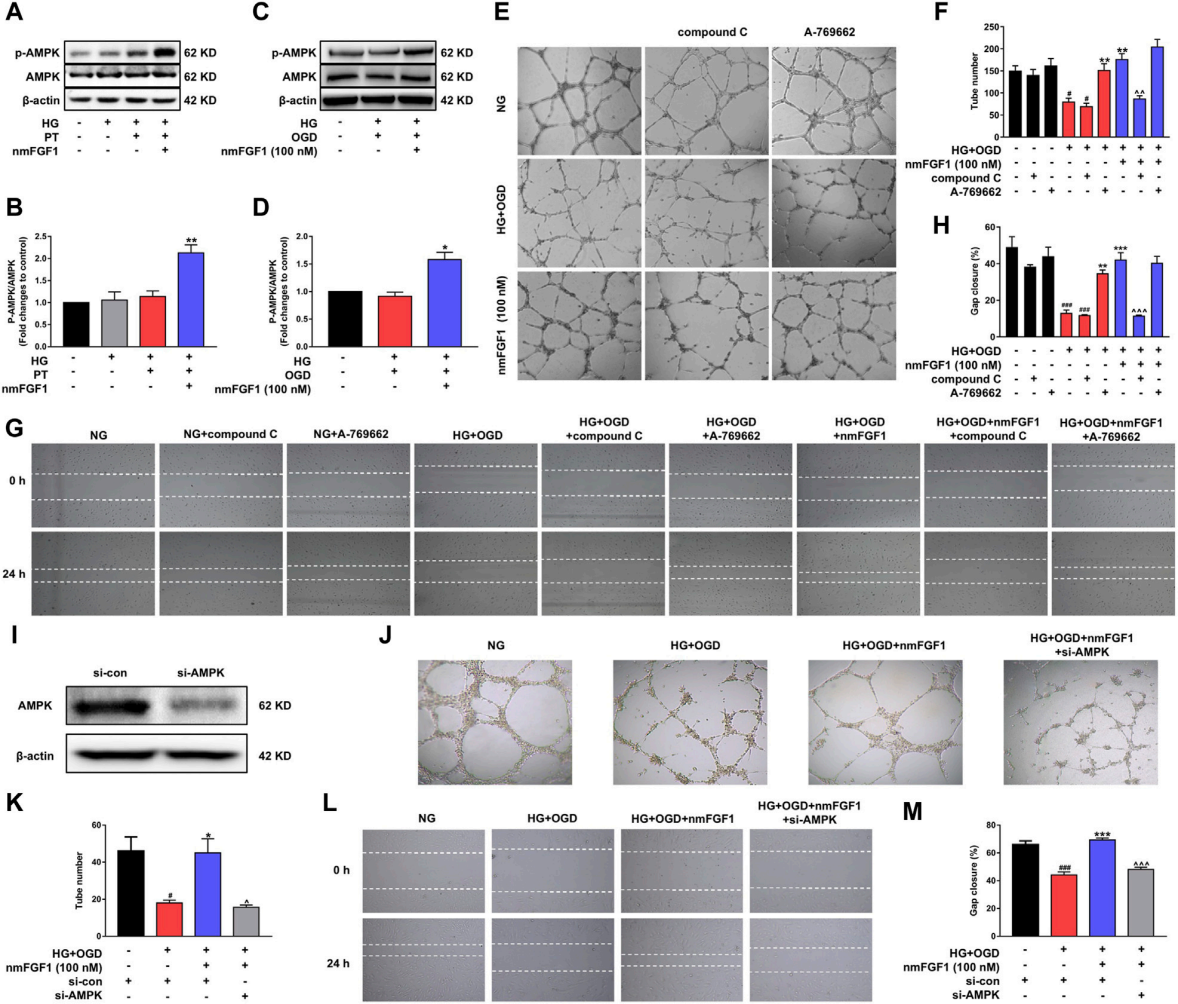
NmFGF1 promoted tube formation and migration of HBMEC in HG + OGD-treated HBMEC cells by activating the AMPK signaling pathway. **(A)** Western blotting analysis of *p*-AMPK and AMPK levels in a mouse model of diabetic stroke treated with or without nmFGF1. β-actin was used as a loading control. **(B)** The ratio of *p*-AMPK/AMPK. **(C)** The levels of *p*-AMPK and AMPK in HBMEC cells, as determined by western blotting. **(D)** The ratio of *p*-AMPK/AMPK in HBMECs, as quantified by image software. **(E)** Cells treated with AMPK agonist or inhibitor. AMPK agonist, A-769662, 10 nM; AMPK inhibitor, compound C, 5 μM. Tube formation was observed by phase contrast microscopy, original magnification: ×40. **(F)** The number of tubes formed in HBMECs treated with AMPK agonist or inhibitor. **(G)** The migration ability of HG + OGD-cultured HBMECs treated with an AMPK agonist or inhibitor. Images of the wound areas were observed by phase-contrast microscopy. A-769662, 10 nM, compound C, 5 μM; Original magnification: ×40. **(H)** The migration ratio was calculated using Image Pro Plus software. **(I)** AMPK expression was silenced by the transfection of siRNA. **(J)** The effect of AMPK silencing on tube formation. Original magnification: ×40. **(K)** Quantitative analysis of tube formation. **(L)** Wounding healing migration assay of cells in which AMPK had been silenced by siRNA. Original magnification: ×40. (M) Quantitative analysis of migration ratio (*n* = 3). Data are presented as means ± SEM. ^#^ *p* < 0.05, ^##^ *p* < 0.01, ^###^ *p* < 0.001 vs NG group; ^*^ *p* < 0.05, ^**^ *p* < 0.01, ^***^ *p* < 0.001 vs HG + OGD or HG + PT group; ^^^ *p* < 0.05, ^^^^ *p* < 0.01, ^^^^^ *p* < 0.001 vs HG + OGD + nmFGF1 group.

The authors apologize for this error and state that this does not change the scientific conclusions of the article in any way. The original article has been updated.

